# Metabolomics reveals soluble epoxide hydrolase as a therapeutic target for high-sucrose diet-mediated gut barrier dysfunction

**DOI:** 10.1073/pnas.2409841121

**Published:** 2024-11-18

**Authors:** Ai-Zhi Lin, Xian Fu, Qing Jiang, Xue Zhou, Sung Hee Hwang, Hou-Hua Yin, Kai-Di Ni, Qing-Jin Pan, Xin He, Ling-Tong Zhang, Yi-Wen Meng, Ya-Nan Liu, Bruce D. Hammock, Jun-Yan Liu

**Affiliations:** ^a^Department of Anesthesia of the Second Affiliated Hospital and CNTTI of College of Pharmacy, Chongqing Medical University, Chongqing 400016, China; ^b^Basic Medicine Research and Innovation Center for Novel Target and Therapeutic Intervention, Ministry of Education, Chongqing 400016, China; ^c^Department of Entomology and Nematology, University of California, Davis, CA 95616; ^d^Comprehensive Cancer Center, University of California, Davis, CA 95616

**Keywords:** Claudins, epoxyeicosatrienoic acid, high sucrose diet, metabolomics, soluble epoxide hydrolase

## Abstract

Highsucrose diet (HSD) was reported as a causative factor for multiorgan injuries. The underlying mechanisms and therapeutic strategies remain largely uncharted. In the present study, by using a metabolomics approach, we identified the soluble epoxide hydrolase (sEH) as a therapeutic target for HSD-mediated gut barrier dysfunction. Specifically, 16-week feeding on an HSD caused gut barrier dysfunction, such as colon inflammation and tight junction impairment in a murine model. A metabolomics analysis of mouse colon tissue showed a decrease in the 5(6)-epoxyeicosatrienoic acid [5(6)-EET] level and an increase in soluble epoxide hydrolase, which is related to HSD-mediated injuries to the gut barrier. The mice treated with a chemical inhibitor of sEH and the mice with genetic intervention by intestinal-specific knockout of the sEH gene significantly attenuated HSD-caused intestinal injuries by reducing HSD-mediated colon inflammation and improving the impaired tight junction caused by an HSD. Further, in vitro studies showed that treatment with 5(6)-EET, but not its hydrolytic product 5,6-dihydroxyeicosatrienoic acid (5,6-DiHET), significantly ablated high sucrose-caused intestinal epithelial inflammation and impaired tight junction. Additionally, 5(6)-EET is anti-inflammatory and improves gut epithelial tight junction while 5,6-DiHET cannot do so. This study presents an underlying mechanism of and a therapeutic strategy for the gut barrier dysfunction caused by an HSD.

## Significance

Feeding mice with a high-sucrose diet (HSD) resulted in colon inflammation and impaired gut barrier tight junction, as well as decreased colon level of 5(6)-epoxyeicosatrienoic acid. This is associated with an increase in its metabolic enzyme, soluble epoxide hydrolase (sEH). Reduction of sEH by both chemical intervention and intestinal-specific knockout of sEH significantly attenuated HSD-mediated gut barrier dysfunction. In vitro studies revealed that treatment with high sucrose led to inflammation in the intestinal epithelia and impaired tight junction. Treatment with 5(6)-epoxyeicosatrienoic acid ablated high sucrose-mediated cellular inflammation and improved tight junctions impaired by high sucrose. The present study gains insights into the pathology and pharmacology of HSD-caused gut barrier dysfunction.

The impact of diets on health has been gaining increasing attention worldwide (1–5). High sucrose (HS), together with high fat, is a typical feature of the Western diet. High-sucrose diet (HSD), including food and beverages rich in sucrose, has been extensively found as a risk factor for a variety of illnesses, such as obesity (6, 7), insulin resistance and glucose intolerance (8, 9), fatty liver (10), systemic and cerebral inflammation (11), and many other chronic diseases (7). In addition, HSD usually leads to additive or synergetic detrimental effects of a high-fat diet mainly because the liberated fructose stimulates the de novo synthesis of fatty acids (12). However, the molecular mechanisms underlying HSD-mediated organ injuries remain largely unknown. In the past four decades, while HSD exposure in high-income countries kept slowly increasing, HSD exposure in low-income and middle-income countries has been markedly increasing along with the accrual export of Western economy, culture, and lifestyles (7). Therefore, there is an urgent need to explore the mechanisms underlying HSD-mediated multiorgan injuries and potential intervention targets. Accordingly, metabolomics, proteomics, and transcriptomics, as well as other innovative techniques, have been employed in the above-mentioned studies (13–16).

Metabolomics is an effective approach to investigate the changes in metabolome caused by multiple endogenous factors and various exogenous factors, including but not limited to genetic mutations, epigenetic modifications, aging, environmental exposure, diet and nutrients, diseases, drugs, and lifestyles (17, 18). Metabolomics has been widely used to investigate pathogenic mechanisms, diagnostic markers, and intervention targets (19–22). Recently, by adopting some advanced techniques, metabolic flux, single-cell metabolomics, and spatial metabolomics were developed as a new generation of metabolomics techniques to enhance the power and performance of traditional metabolomics (23–26). Notably, we have used both nontargeted metabolomics and targeted metabolomics methods to investigate the potential intervention targets or biomarkers for acute kidney disease, obesity-related kidney injury, chronic kidney disease, gouty arthritis, and colon cancers (17, 27–30). In particular, a targeted metabolomics approach to the metabolism of polyunsaturated fatty acids (PUFAs), including linoleic acid, alpha-linolenic acid, arachidonic acid, eicosapentaenoic acid (EPA), and docosahexaenoic acid (DHA), was extensively employed to explore the mechanisms underlying PUFAs metabolism-driven pathogenic and pharmacological process (27–30). Most organ and tissue injuries are associated with inflammation, which is closely associated with the metabolism of PUFAs and particularly the metabolism of arachidonic acid. Therefore, in the present study, we used the established targeted metabolomics approach to investigate the role of PUFAs’s metabolism in HSD-mediated gut barrier injury. Here, we report that soluble epoxide hydrolase (sEH), the metabolic enzyme mediating the transformation of epoxide fatty acids (EpFAs) derived from PUFAs by cytochrome P450, to form conversely or less active vicinal diols, is a prospective intervention target for prophylactic and therapeutic treatments of HSD-mediated gut barrier dysfunction.

## Results

### HSD did not Cause an Excess Gain of Body Weight in a Murine Model.

**Fig. 1.**
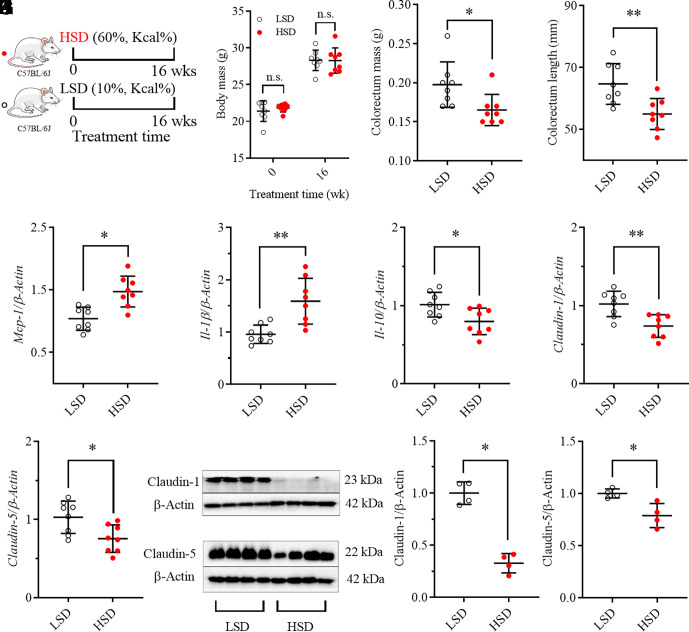
HSD caused colorectum inflammation and injury to gut barrier integrity in a murine model. (*A*) A brief scheme of the experimental design; mice fed with an HSD caused no excess weight gain (*B*) but significantly decreased colorectum mass (*C*) and length (*D*). Mice fed with an HSD increased inflammatory *Mcp-1* (*E*) and *Il-1β* (*F*) but decreased anti-inflammatory *Il-10* (*G*), as well as decreased Claudin-1 and Claudin-5 at mRNA (*H* and *I*) and protein levels (*J*–*L*). Data represent mean ± SD (N = 8 or 4). Statistical difference was determined by a two-tailed noncompartmental test with the Mann–Whitney test (*0.01 < *P* ≤ 0.05, **0.001 < *P* ≤ 0.01).

As illustrated in Fig. 1*B*, the mice fed with an HSD did not experience excess weight gain after 16 wks of feeding when compared with the ones fed with a low sucrose diet (LSD).

### HSD Caused Colorectal Inflammation in a Murine Model.

Feeding the mice with an HSD led to significant decreases in colorectum mass (Fig. 1*C*) and length (Fig. 1*D*) when compared with mice fed with an LSD. In addition, HSD feeding to mice resulted in significant increases in inflammatory cytokines *Mcp-1* (Fig. 1*E*) and *Il-1β* (Fig. 1*F*), and significant reduction in the anti-inflammatory *Il-10* (Fig. 1*G*) at mRNA levels in colorectum tissue when compared with an LSD feeding. In addition, pathological analysis revealed that the mice fed with HSD resulted in obvious inflammatory cell infiltration (*SI Appendix*, Fig. S1).

### HSD Caused Injury to Gut Barrier Dysfunction in a Murine Model.

HSD feeding to mice for 16 wks caused significantly lower Claudin-1 and Claudin-5, the markers of intestine permeability, at the mRNA (Fig. 1 *H* and *I*) and protein (Fig. 1 *J–L* and *SI Appendix*, Fig. S2) levels in colorectum tissue than an LSD did.

### Targeted Metabolomics Revealed the Upregulation of Colorectum sEH in the Mice Fed on an HSD.

**Fig. 2.**
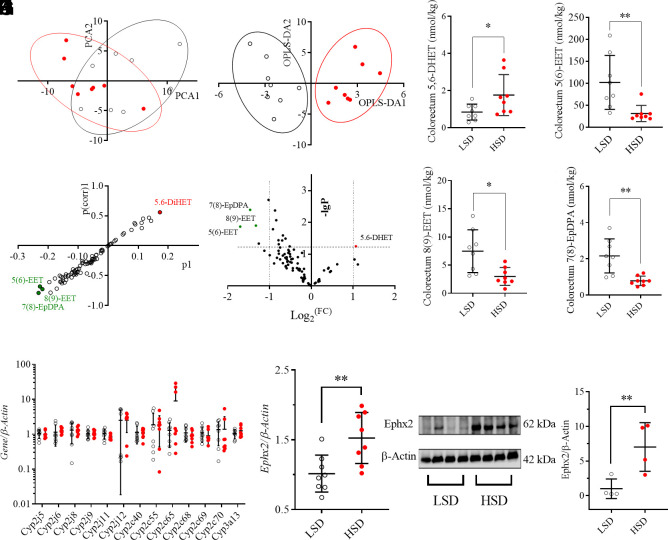
HSD caused upregulation of soluble epoxide hydrolase (sEH, coding gene *Ephx2*). (*A*) Two-dimensional PCA score plots show visual difference of the mice fed with HSD (red dots) from the controls (LSD, unfilled cycles), R2X=0.706, Q2=0.413; (*B*) Two-dimensional OPLS-DA score plots show visual difference of the mice fed with HSD (red dots) from the controls (LSD, unfilled cycles), R2X = 0.605, Q2 = 0.527; The S-plots (*C*) and volcano plot (*D*) of the OPLS-DA model for the processed data; Colorectum levels of 5,6-DiHET (*E*), 5(6)-EET (*F*), 8(9)-EET (*G*), and 7(8)-EpDPA (*H*) for the mice fed with HSD and LSD; HSD feeding caused slight modification of *Cyp2cs*, Cyp3a13, and *Cyp2js* (*I*) but a significant increase in Ephx2 at mRNA (*J*) and protein (*K* and *L*) levels. Data represent mean ± SD (N = 8 or 4). Statistical difference was determined by a two-tailed noncompartmental test with the Mann–Whitney test (*0.01 < *P* ≤ 0.05, **0.001 < *P* ≤ 0.01).

An established targeted metabolomics analysis of murine colorectum tissue from the mice fed on an HSD and LSD recorded 85 oxylipins (*SI Appendix*, Table S1) in the analyzed samples. An unsupervised multivariate method principal component analysis (PCA) was employed to process the recorded concentration, resulting in a visual difference between the mice fed on an HSD from those with LSD (Fig. 2*A*), by which the first three components of the PCA took 70.6% of the variation of the data. Then orthogonal partial least squares-discriminant analysis (OPLS-DA), a supervised multivariate method, was employed to process the data. Two-dimensional score plot, constructed by one predictive component and two orthogonal components model, led to an obvious visual separation of the mice fed on an HSD from the ones on an LSD (Fig. 2*B*). Interestingly, both S-plot (Fig. 2*C*) and a volcano plot (Fig. 2*D*) identified the decreases in three EpFAs, including 5(6)-EET, 8(9)-EET, and 7(8)-EpDPA, and increase in 5,6-DiHET in the colorectum tissue from the mice fed on an HSD as the major factors leading to the separation of the mice fed on an HSD from the ones with an LSD. The significant differences of these four oxylipins between the two groups were further supported by a noncompartmental test with the Mann–Whitney test (Fig. 2 *E*–*H*). Because EpFAs were the products of PUFAs in the presence of *Cyp2cs* and *Cyp2js* and were further metabolized by sEH, colorectum expression of these genes at mRNA levels was quantitatively analyzed. As illustrated in Fig. 2*I*, HSD feeding caused nonsignificant changes in *Cyp2cs, Cyp2js and Cyp3a13*. It should be noted that data of *Cyp2c29/37/38/39/44* are not presented because they are expressed over 10-fold lower than the illustrated ones. However, sEH (coding gene, *Ephx2*) was up-regulated in the colorectum tissue of the mice fed on an HSD when compared with those of LSD at both mRNA (Fig. 2*J*) and protein (Fig. 2 *K* and *L*) levels.

### Intestinal sEH Is Involved in the Metabolism of 5(6)-EET and 14(15)-EET.

To test whether intestinal sEH is involved in the metabolism of 5(6)-EET, we tested the degradation of 5(6)-EET and 14(15)-EET in cell culture media (without enzymes), normal LoVo cells (that express sEH), and LoVo cells with knockout of sEH. The knockout efficacy of sEH was supported by western blot analysis (*SI Appendix*, Fig. S3 *A* and *B*). As shown in *SI Appendix*, Fig. S3 *C*–*E*, when in the media with no enzyme, unlike 14(15)-EET being stable through the tested time (10 h), 5(6)-EET was degraded rapidly. However, in the presence of a cellular system with enzymes, knockout of *sEH* reduced the degradation significantly of 5(6)-EET and 14(15)-EET as well.

### Treatment with an sEH Inhibitor Attenuated HSD-Mediated Colorectal Inflammation and Gut Barrier Dysfunction.

**Fig. 3.**
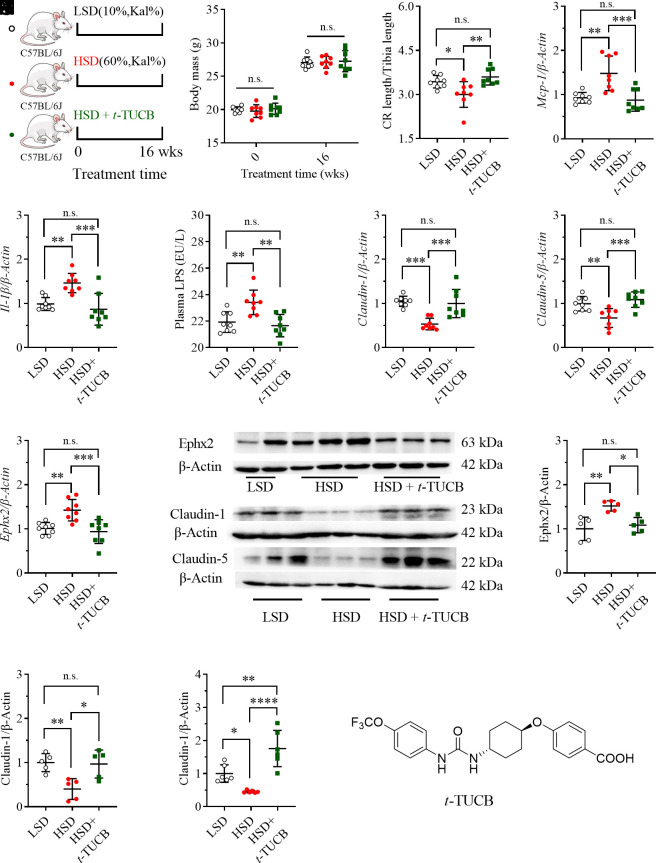
Inhibition of sEH with an sEH inhibitor attenuated HSD-mediated injury to gut barrier integrity. (*A*) A brief scheme of the experimental design; (*B*) Mice fed with an HSD with or without *t*-TUCB did not cause excessive body gain; Treatment with the sEH inhibitor *t*-TUCB attenuated HSD-mediated reduction in the ratio of colorectal length to tibia length (*C*), increase in plasma LPS level (*D*), increases in colon *Mcp-1* (*E*) and *Il-1β* (*F*), decreases in colon claudin-1 and claudin-5 and increase in colon sEH at mRNA levels (*G–I*) and corresponding changes at protein levels (*J*, *K*, *L*, and *M*), as well as increase in colon sEH at mRNA (*I*) and protein levels (*J* and *K*); (*N*) Structure of *t*-TUCB, an inhibitor of sEH. Data represent mean ± SD (N = 8 or 5 to 6). Statistical difference was determined by ANOVA followed by a Bonferroni’s (variance homogeneity) or Dunnett’s (variance heterogeneity) post hoc comparison test (* 0.01 < *P* ≤ 0.05, **0.001< *P* ≤ 0.01, ***0.0001< *P* ≤ 0.001, **** *P* ≤ 0.0001).

To test whether sEH is an intervention target for HSD-caused colorectal inflammation and injury, a sEH inhibitor *t*-TUCB (structure shown as Fig. 3*N*) was provided in drinking water to the mice fed with an FSD (Fig. 3*A*). We used *t*-TUCB in the present study based on its potent inhibitory activity against murine sEH and a satisfactory pharmacokinetic profile in mice (31). As expected, the mice treated with an HSD with or without *t*-TUCB slightly changed the mouse’s body mass (Fig. 3*B*). The treatment with *t*-TUCB significantly reversed HSD-mediated decrease in relative colorectal length (Fig. 3*C*), and inflammatory *Mcp-1* (Fig. 3*D*) and *Il-1β* (Fig. 3*E*) in colorectal tissue. Feeding the mice with HSD led to a significantly increased plasma level of LPS, which was significantly reduced by *t*-TUCB treatment (Fig. 3*F*). HSD-mediated decreases in colorectal Claudin-1 and Claudin-5 at mRNA and protein levels were significantly up-regulated by *t*-TUCB treatment (Fig. 3 *G*, *H*, *J*, *L*, and *M* and *SI Appendix*, Fig. S6). The action of *t*-TUCB against sEH at both mRNA and protein levels is shown in Fig. 3 *I*–*K*, which was also supported by the increase in the sum of colorectum EET levels by TPPU treatment (*SI Appendix*, Fig. S4). In addition, pathological analysis revealed that treatment of *t*-TUCB attenuated HSD-mediated inflammatory cell infiltration in the colon tissue (*SI Appendix*, Fig. S5).

### Intestinal-Specific Knockout of sEH Attenuated HSD-Mediated Colorectal Inflammation and Gut Barrier Dysfunction.

**Fig. 4.**
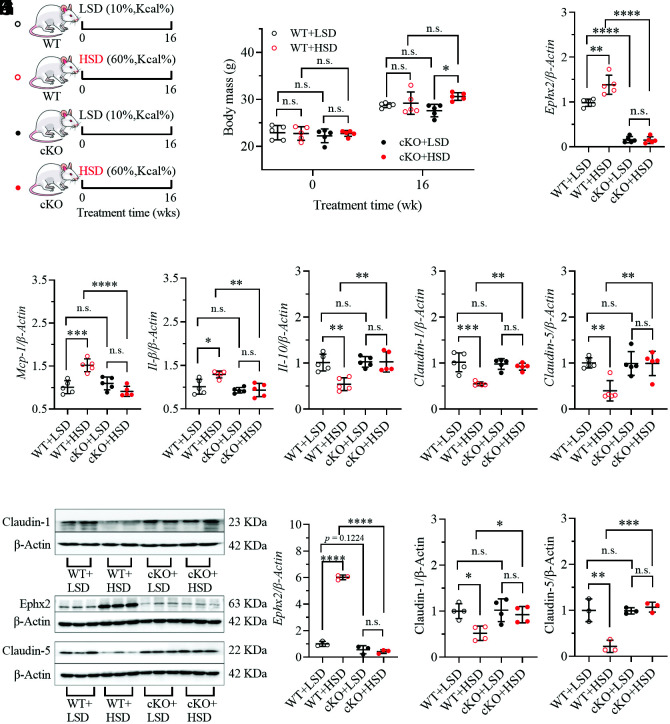
Intestinal epithelial-specific knockout (cKO) of sEH attenuates HSD-mediated injury to the gut barrier. (*A*) A brief scheme of the experimental design; (*B*) Both cKO mice and their wildtype (WT) littermates fed on an HSD did not cause excessive body gain; (*C*) An HSD feeding resulted in upregulation of intestinal *Ephx2* in WT mice while slight change in *Ephx2* cKO mice; An HSD feeding resulted in significance increases in intestinal *Mcp-1* (*D*) and *IL-1β* (*E*), as well as significant decreases in intestinal *IL-10* (*F*), *Claudin-1* (*G*), and *Claudin-5* (*H*) in WT mice. However, these significant changes in the intestine caused by an HSD feeding were reduced by conditional knockout of *Ephx2* in the intestine; (*I*) Conditional knockout of *Ephx2* in the intestine diminished the significant increase in Ephx2 and decreases in Claudin-1 and Claudin-5 caused by an HSD feeding in the WT littermates; The quantitative analyses of band density were presented as (*J*–*L*). Data represent mean ± SD (N = 3 to 5). Statistical difference was determined by ANOVA followed by a Bonferroni’s (variance homogeneity) or Dunnett’s (variance heterogeneity) post hoc comparison test (*0.01 < *P* ≤ 0.05, **0.001 < *P* ≤ 0.01, ***0.0001 < *P* ≤ 0.001, ****P ≤ 0.0001).

Conditional (intestinal-specific) knockout (cKO) of sEH was identified by PCR analysis of the mouse toe tips (*SI Appendix*, Fig. S7), which was further supported by the lower mRNA and protein levels of sEH in intestinal tissue from cKO mice fed on an LSD than the WT mice on an LSD (Fig. 4 *C*, *I*, and *J*). Similar to *t*-TUCB treatment, cKO mice fed with an HSD resulted in a dramatic decrease in mRNA and protein levels of sEH when compared with WT mice fed with an HSD (Fig. 4 *C*, *I*, and *J*). Therefore, it could be expected that cKO of sEH fed with an HSD or LSD led to nonsignificant change in mice body weight when compared with the WT mice treated with an LSD or HSD, exactly as shown in Fig. 4*B*. However, cKO of sEH significantly attenuated HSD-mediated increase in intestinal *Mcp*-1 (Fig. 4*D*), *Il-1β* (Fig. 4*E*), and decrease in intestinal *Il-10* (Fig. 4*F*) in WT mice. Again, cKO of sEH significantly up-regulated the HSD-mediated decrease in intestinal Claudin-1 and Claudin-5 at mRNA and protein levels (Fig. 4 *G*, *H*–*L* and *SI Appendix*, Fig. S9). Histological analyses of colon tissue also indicated that cKO of sEH attenuated HSD-mediated colon inflammation (*SI Appendix*, Fig. S8).

### Treatment with High Sucrose (HS) Caused Injury to Intestinal Epithelial Cells.

**Fig. 5.**
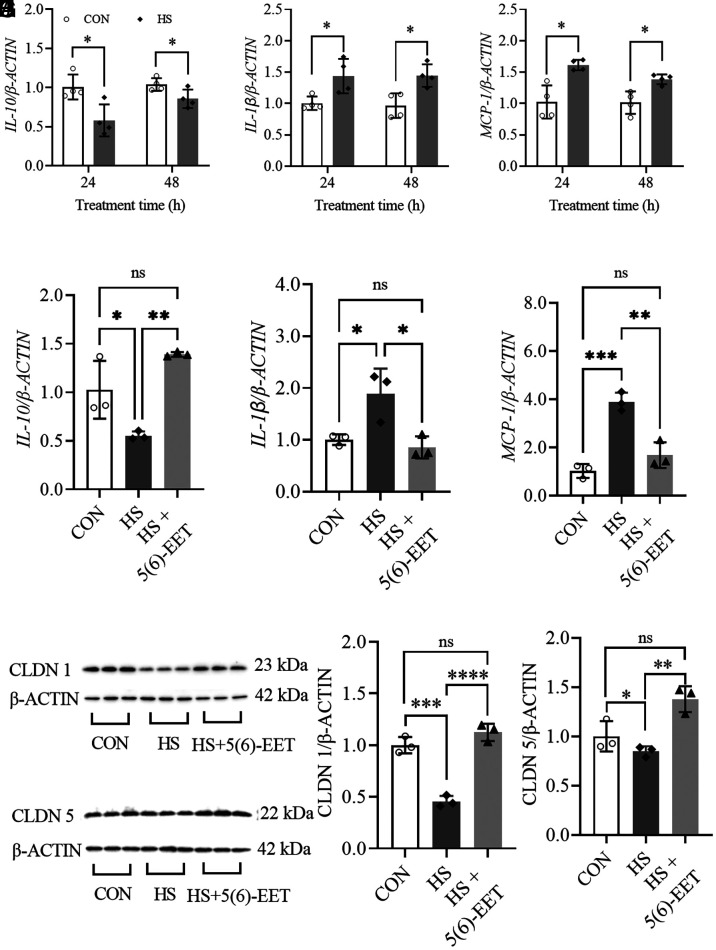
5(6)-EET attenuates high-sucrose (HS)-mediated injury to intestinal epithelial cells. The treatment of HS resulted decrease in *IL-10* (*A*), and an increase in *IL-1β* (*B*) and *MCP-1* (*C*) in LoVo cells in a time-relevant manner. The treatment of 5(6)-EET (100 nM) significantly reversed the HS-mediated decrease in *IL-10* (*D*), and increase in *IL-1β* (*E*) and *MCP-1* (*F*). The treatment of 5(6)-EET (100 nM) significantly ablated HS-mediated decrease in Claudin-1 and 5 (*G*–*J*). (*H*) and (*J*) are the quantitative analysis results of band density presented in (*G*) and (*I*), respectively. Data represent mean ± SD (N = 3 to 4). The statistical difference between the two groups was determined by a two-tailed noncompartmental test with the Mann–Whitney test, while the statistical difference among three groups was determined by ANOVA followed by a Bonferroni’s (variance homogeneity) or Dunnett’s (variance heterogeneity) post hoc comparison test (*0.01 < *P* ≤ 0.05, **0.001 < *P* ≤ 0.01, ***0.0001< *P* ≤ 0.001, ****P ≤ 0.0001).

Treatment of human colon epithelial cells (LoVo) with high sucrose (HS) resulted in a significant decrease in cell *IL-10* (Fig. 5*A*), and a significant increase in cell *IL-1β* (Fig. 5*B*) and *MCP-1* (Fig. 5*C*) at mRNA levels. It should be noted that, at each treatment time, we took the control cells as the baseline for the corresponding HS-treated cells, although the mRNA levels of all three cytokines increased along with the treatment time. In addition, treatment of HS also caused a significant decrease in CLAUDIN 1 (CLDN1) and CLAUDIN 5 (CLDN5) (Fig. 5 *G*–*I*) at the protein level.

### 5(6)-EET but not 5,6-DiHET Attenuated HS-Mediated Injury to Intestinal Epithelial Cells.

As illustrated in Fig. 5 *D*–*F*, treatment of 5(6)-EET significantly reversed the HS-mediated decrease in cell *IL-10* and an increase in *IL-1β* and *MCP-1* at mRNA levels. In addition, 5(6)-EET treatment increased the HS-mediated decrease in cell CLAUDIN-1 and 5 at the protein level (Fig. 5 *G*–*J*). However, treatment of 5,6-DiHET at the same concentration of 5(6)-DiHET failed in reversing HS-induced damages to the tested cells (*SI Appendix*, Fig. S10).

### 5(6)-EET but not 5,6-DiHET Is Anti-Inflammatory and Increases Tight Junction for Intestinal Epithelial Cells.

Treatment of the human colon epithelial cells (NCM460) with 5(6)EET resulted in a decrease in cell *IL-1β* and *CLDN1* (*SI Appendix*, Figs. S11*A*, and S11*B*) at mRNA levels in a time-dependent manner. In addition, 5(6)-EET treatment resulted in a time-dependent increase in cell CLDN1 (*SI Appendix*, Fig. S11 *E*–*G*) at protein levels. In contrast, the treatment of NCM460 cells with the same dose of 5,6-DiHET, the sEH-mediated product of 5(6)-EET, slightly modified the cell mRNA levels of *IL-1β* and *CLDN1* (*SI Appendix*, Figs. S11*C* and S8*D*), and protein levels of CLDN1 (*SI Appendix*, Fig. S11*H*). These changes have been shown to correlate 5(6)-EET with the resolution of inflammation.

## Discussion

This study disclosed that even under healthy conditions, continuous consumption of an HSD resulted in injuries to the intestinal barrier, including intestinal inflammation and impaired gut barrier tight junction, together with an upregulation of sEH, which was subsequently showed to be a potential therapeutic target for HSD-mediated gut injuries. HSD-mediated intestinal inflammation was evidenced by HSD-caused increase in proinflammatory cytokine *IL-1β* and *Mcp-1* and decrease in anti-inflammatory *IL-10* at mRNA levels (Fig. 1 *E*–*G*). As expected, HSD-caused upregulation of *Mcp-1* and *IL-1β* was also observed in the other two animal experiments (Figs. 3 *D* and *E* and 4 *D*–*E*). Unfortunately, HSD feeding led to a slight decrease in *IL-10* in the mice fed with an HSD compared with the ones with an LSD in the second animal experiment (data now shown), perhaps due to the different batches of the mice in the two experiments. However, a significant decrease in *IL-10* was consistently observed in WT mice feeding with an HSD in the third animal experiment (Fig. 4*F*). In addition, colorectal inflammation caused by an HSD feeding is also supported by shortened colon length caused by HSD consumption (Fig. 1*D*). Meanwhile, decrease in colorectal Claudin-1 and Claudin-5 at both mRNA and protein levels were consistently observed in the mice fed with an HSD compared with the mice with an LSD in all three animal experiments (Figs. 1 *H*–*L*, 3 *G*, *H*, *J*, *L*, and *M*, 4 *G*–*I*, *K*, and *L*), indicative of the reduced gut barrier tight junction. In addition, the impaired intestinal barrier in the mice fed with a HSD was also evidenced by increased plasma level of LPS, which is a marker of intestinal permeability (Fig. 3*F*). These results collectively support that long-term feeding of HSD caused gut barrier dysfunction. The gut barrier dysfunction caused by an HSD in this study is also supported by several previously reported studies (32–34). In addition, sucrose will be hydrolyzed to form equivalent fructose and glucose in the mammal body in the presence of sucrase-isomaltase. Many scientists directly investigated the side effects of excessive consumption of fructose and/or glucose. Consistently, both a high-fructose diet and a high-glucose diet were reported to induce impaired gut barrier (35–37).

A targeted metabolomics study of colon oxylipins demonstrated the critical role of CYP/sEH-mediated EpFAs and corresponding diols [5(6)-EET, 8(9)-EET, 7(8)-EpDPA, and 5,6-DiHET] in HSD-mediated gut barrier dysfunction (Fig. 2 *A*–*D*). CYPs were excluded as the causative factors for the changes of EpFAs and diols because they were slightly modified by HSD feeding (Fig. 2*I*). In contrast, sEH was found to be increased in colon tissue at both mRNA and protein levels (Fig. 2 *J*–*L*), which was in agreement with the HSD-caused increase in sEH in the latter two animal experiments as shown in Figs. 3 and 4, respectively. Subsequently, sEH was found an intervention target for HSD-mediated gut barrier dysfunction because inhibition of sEH with a chemical inhibitor significantly ameliorated HSD-caused reduced gut barrier tight junction and colon inflammation (Fig. 3 *C*–*M*), which was further in accordance with the findings obtained from feeding the mice with a conditional knockout of sEH in the gut epithelia with an HSD (Fig. 4). In addition, a sEH was also reported a therapeutic target for high-fat diet-caused colonic inflammation and gut barrier dysfunction (38), which further supports the findings in this study.

To explore whether reduced colon levels of EpFAs are the causative factor for gut barrier dysfunction, we selected 5(6)-EET and 5,6-DiHET for further study because they were key mediators contributing to the separation of the mice treated with an HSD and LSD (Fig. 2 *B*–*D*). Treatment of HS-induced LoVo cells with 5(6)-EET or 5,6-DiHET demonstrated that 5(6)-EET is more active in reversing HS-induced injury to tested cells (Fig. 5 and *SI Appendix*, Fig. S10), which was further consistent with the findings from the treatment of human gut epithelia (NM460) with 5(6)-EET and 5,6-DiHET (*SI Appendix*, Fig. S11). Taken together, these in vitro results explain the decreased colon level of 5(6)-EET and increased colon level of 5,6-DiHET contributed greatly to the gut barrier dysfunction caused by HSD feeding. Administration of DiHETs to mice resulted in murine colon inflammation and a decreased colon level of Claudin-5 (39). This study disclosed HSD feeding caused an increase in 5,6-DiHET, in the murine colon, which also supports that the increase in 5,6-DiHET could cause injury to the gut barrier. However, the increased colon levels caused by HSD were only found for 5,6-DiHET but not 14,15-, 11,12-, and 8,9-DiHET in this study, indicating the complexity of the CYP/sEH axis in mediating the metabolism of AA because DiHETs could be further metabolized (28).

One could concern that 5(6)-EET is chemically metabolized to form 5,6-DiHET lactone directly without the involvement of sEH (40). As shown in *SI Appendix*, Fig. S3, when in cell culture media without enzymes, 14(15)-EET was stable while 5(6)-EET degraded rapidly, indicating that 5(6)-EET is degraded at least in part nonenzymatically, which is consistent with the previously reported study about 5(6)-EET (40). However, in a cellular system containing sEH, the degradation of 5(6)-EET, was similar to that of 14(15)-EET, was slowed down in the knockout of sEH, indicating 5(6)-EET, similar to 14(15)-EET, could be also metabolized by sEH. Our study revealed that 5(6)-EET could be metabolized through sEH, in parallel to the predominantly nonenzymatic pathways.

In addition, increased plasma level of LPS in the mice fed on a HSD convinced impaired gut barrier function by increased gut barrier permeability. Because gut microbiota is the major source of LPS, an HSD feeding may cause gut dysbiosis to drive the impaired gut barrier and up-regulate sEH (41), the details of which need further investigation. We also knew that sugar consumption varies between individuals and throughout the world. Sucrose is the most common sugar in the diets and beverages with the major source of cane sugar. Corn syrup is another common sugar used in diets and beverages, which contains varying amounts of glucose, fructose, maltose, and many oligosaccharides. A sucrose can be broken to form equal a fructose and a glucose in the body. Sucrose, glucose, and fructose have similar effects on health, but not always the same (42, 43). This study focused solely on sucrose instead of a comparison study of the three common sugars.

In brief, this study provides pathological insights into HSD-mediated organ injuries, but also proposes an intervention method to attenuate HSD-mediated gut barrier dysfunction. In addition, this study extends our understanding of the potential application of sEH inhibitors, perhaps a diet rich in natural sEH inhibitors, for example, capsaicin, macamides, and their source materials (44, 45), could benefit people with a fondness for an HSD.

## Materials and Methods

All animal experiments were conducted in line with the protocols approved by the Animal Use and Care Committee of Chongqing Medical University. The mice accessed to LSD or HSD and water *ad-lib* for 16 wk. LSD (10% kcal sucrose, AIN93G) and HSD (60% kcal sucrose, D12329) were purchased from Wuxi Fanbo Biotechnology Co., Ltd. (Wuxi, China), which were manufactured according to the formula and protocols of Research Diets, Inc. (New Brunswick, NJ). *t*-TUCB was administered in drinking water (10 mg/L). The *Ephx2^flox/flox^* mice and Villin-Cre mice were commercially acquired from the Shanghai Model Organisms Center, Inc (Shanghai, China). An *Ephx2 flox* heterozygous mouse was crossed with a Villin-Cre mouse to generate *Ephx2 flox/flox*;Villin-Cre (cKO) mice for further experiment. The group information is presented in Figs. 1–4. The full information of materials, animal and cellular protocols, and analytical protocols is provided in online *SI Appendix*.

## Supplementary Material

Appendix 01 (PDF)

## Data Availability

All data and materials are included in the article and/or *SI Appendix*.
