# Supplementary material for: Metabolomics reveals soluble epoxide hydrolase as a therapeutic target for high-sucrose diet-mediated gut barrier dysfunction
Source: Proc Natl Acad Sci U S A. 2024 Nov 18;121(48):e2409841121. doi: 10.1073/pnas.2409841121 (PMC11621843; doi:10.1073/pnas.2409841121)
Supplement: Supplementary file 1 — Appendix 01 (PDF) [file pnas.2409841121.sapp.pdf]

PNAS

Supplementary Information for

Metabolomics reveals soluble epoxide hydrolase as a therapeutic target for high-sucrose diet-mediated gut barrier dysfunction

Ai-Zhi Lin, Xian Fu, Qing Jiang, Xue Zhou, Sung Hee Hwang, Hou-Hua Yin, Kai-Di Ni, Qing-Jin Pan, Xin He, Ling-Tong Zhang, Yi-Wen Meng, Ya-Nan Liu, Bruce D. Hammock, Jun-Yan Liu

Corresponding author: Jun-Yan Liu and Bruce D. Hammock

Email: jyliu@cqmu.edu.cn and bdhammock@ucdavis.edu

This PDF file includes:

Supplementary text

References for SI reference citations

Figs. S1 to S12

Tables S1 to S5

Supplementary text

Materials and Methods

The oxylipin standards in the oxylipin quantitation method, 5(6)-EET, 5,6-DiHET, and 14(15)-EET tested in cell models of this project, as well as the antibody for sEH (used in the most cases except Fig. S2), were purchased from a local distributor of Cayman Chemical (Ann Arbor, MI) in Shanghai, China. *Trans*-1-(4-trifluoro-methoxy-phenyl)-3-((1-yl-ureido)-cyclohexyloxy)-benzoic acid; (*t*-TUCB) was synthesized by the Hammock Laboratory according to the previously reported procedure (1). The antibodies for β-actin, Claudin-1, Claudin-5, and Goat Anti-Rabbit IgG/HRP were purchased from ZenBio Inc. (Chengdu, China). The antibodies for HSP90 and sEH (used in Fig. S2) were purchased from Proteintech Group (Wuhan, China). The primers for quantitative real-time PCR were manufactured by Sangon Biotech Co. Ltd. (Shanghai, China) with the sequences presented in SI Appendix Table S3. The colon epithelial cell (NCM460) was purchased from Shanghai Guyan Industrial Co., Ltd. (Shanghai, China) and the colorectal cancer cell (LoVo) was purchased from Shanghai Zhong Qiao Xin Zhou Biotechnology Co., Ltd. (Shanghai, China). Polybrene and puromycin were purchased from Beyotime Biotechnology (Shanghai, China)The culture medium RPMI-1640, FBS, and penicillin-streptomycin stock solution were purchased from a local distributor of Thermo Fisher Scientific (Rockford, IL).

Generation of *Ephx2*$^{flox/flox}$ mouse model

Ephx2$^{flox/flox}$ mice were generated by Shanghai Model Organisms Center, Inc. (Shanghai, China). This model was generated by CRISPR/Cas9 technology in C57BL/6J mouse background. To generate the *Ephx2*-flox line, in which exons 4-5 of the *Ephx2* allele are flanked by loxp sites,

four independent guide RNAs targeting *Ephx2* introns 3 and 5 were designed. The *Ephx2* donor vector containing loxp sites flanking exons 4-5 and two homology arms was used as a template. Two gRNAs targeted to intron 3 were 5'-CTAAGTCTCCTCAGTTTACG-3' and 5'-GGAGAGAACCACGTAAACTG-3'. Two gRNAs targeted to intron 5 were 5'-CAAATAATGAGTATATTAAC-3' and 5'-CAGTTAATATACTCATTATT-3'. The donor vector with four gRNAs and Cas9 mRNA was microinjected into C57BL/6J fertilized eggs. The positive founder mice were mated to WT C57BL/6J mice to obtain *Ephx2* flox heterozygous mice. Progeny was screened by PCR for germ-line transmission of the targeted alleles. The primers (P1-P2) used for genotyping the correct 3' homology arm recombination were P1: 5'-ATGTAATCTTAGCACTGGGGA-3' and P2: 5'-GATACTTCAGGTCTTGTGTTGTT-3'. The PCR products were further confirmed by sequencing.

Ephx2 flox heterozygous mice were crossed with Villin-Cre mice to generate *Ephx2* $^{flox/flox}$;Villin-Cre mice. To genotype the *Ephx* alleles, two primers (P1: 5′-ATGTAATCTTAGCACTGGGGA-3′ and P2: 5′-GATACTTCAGGTCTTGTGTTGTT-3′) were used to detect the WT allele (261bp) and the loxp allele (336bp). To genotype the Cre transgene, three primers (F: 5′-GCCTTCTCCTCTAGGCTCGT-3′, R: 5′-TATAGGGCAGAGCTGGAGGA-3′ and R: 5′-AGGCAAATTTTGGTGTACGG-3′) were utilized to amplify a Cre-specific product of 727bp and Internal positive control product 182bp (**Appendix SI Fig. S5**).

Animal protocols

All animal experiments were performed according to the protocols approved by the Animal Use and Care Committee of Chongqing Medical University. Male mice (C57BL/6J, 7-week-old) were purchased from GemPharmtech (Chengdu, China). The mice were housed in a temperature-consistent animal room with a 12-hour light/dark cycle and access to food and water *ad-lib*. An LSD (10% kcal sucrose, AIN93G) and an HSD (60% kcal sucrose, D12329) were purchased from Wuxi Fanbo Biotechnology Co., Ltd. (Wuxi, China), which were manufactured according to the formula and protocols of Research Diets, Inc. (New Brunswick, NJ).

In the first animal experiment, the mice were randomly assigned into two groups (n=8). After one week of accommodation, the mice in one group were fed on an LSD and the other HSD for 16 weeks. The animal's body weight was recorded before the treatment and then once a week. The diet consumption was recorded per cage every week to roughly calculate the average diet consumption/mouse/day (Appendix Fig. S12). The overnight fasted mice were anesthetized by ip injection of pentobarbital sodium (2% m/m in saline) solution with a dose of 0.1 mL/20 g body mass. Blood was collected through the orbital sinus into a clean Eppendorf tube containing a 10 uL antioxidant cocktail (2), which was then centrifuged at × 1500 g for 10 min to get plasma. The plasma sample then was distributed into 500-uL clean Eppendorf tubes as planned. The colon was collected from the connection of cecum and small intestine through the anus. After measuring the colon length, the colon was vertically opened by surgical scissors and rinsed in ice-cold saline. After drying with tissue paper, the colon was then recorded the weight and then cut and distributed in 2-mL cryotubes as planned. All the samples were flashed frozen in liquid nitrogen and then stored under -80°C until analysis.

In the second animal experiment, the mice were assigned into three groups at random, each group containing 8 animals. The mice of the first two groups were treated as those in the first

animal experiment described above. The mice in the third group were fed with an HSD and were simultaneously provided with drinking water (0.2% PEG400) containing *t*-TUCB at a concentration of 10 mg/L. The treatment time and sample collection were as same as those described in the first experiment. The water drunk was recorded per cage every week to evaluate the approximate dose for the mouse as about 2 mg/kg/day.

In the third animal experiment, 7-week-old, male cKO mice and their male littermates were assigned into two groups at random (n = 5 each), respectively. The mice of one group were fed with an LSD and the ones of the second group were fed on an HSD for 16 weeks. The treatment and sample collection were the same as those described for the first animal experiment.

Histological Examination

The colon tissues were prepared for microscopic analysis with H.& E. stain following previously reported protocol (3).

Immunohistochemistry (IHC)

Immunohistochemical (IHC) staining was performed using a two-step IHC detection kit (Elabscience, #E-IR-R217). In brief, paraffin-embedded mouse intestinal tissue sections were deparaffinized and hydrated, and then boiled in a sodium citrate antigen-repair solution for 10 min in a microwave oven. When the solution was cooled to room temperature, sections were incubated with E-IR-R217C (3% H_2O_2) for 15 min at room temperature to inactivate the endogenous enzyme. Next, sections were washed with PBS 3 times for 3 min each, followed by incubation with E-IR-R217A (normal goat serum) at 37°C for 30 min. 30min later, the excess fluid was discarded and the primary antibody (Claudin 1: Proteintech, #28674-1-AP; Claudin 5: Proteintech, #29767-1-AP) were incubated overnight at 4°C. The next day, after washed with PBS, the sections were

incubated with E-IR-R217B (Polyperoxidase-anti-Mouse/Rabbit IgG) for 30 min at room temperature. Subsequently, PBS-washed sections were stained with DAB. The staining was terminated by rinsing with tap water after a positive signal was observed under the microscope, and the sections were then subjected to re-staining, dehydration, transparency and sealing. The positive areas of the IHC were calculated using the Image J software.

Cell culture protocols

The human colon epithelial cells (NCM460) or LoVo were cultured in the associated cell culture medium RPMI-1640 + 10% FBS + 1% penicillin-streptomycin. The cells were maintained in the 10 cm^2 tissue culture flasks at 37℃, in a 5% CO_2 humidified atmosphere. After reaching approximately 80% confluency, the cells were treated with the control or vehicle, sucrose (8 mg/mL) with or without 5(6)-EET or 5,6-DiHET. The concentration of 5(6)-EET, and the culture time were presented in Fig. 5. Ethanol (0.01%, v/v) served as a vehicle. The cells were harvested and prepared for western blot or qPCR analysis. All experiments were performed in multiplicate noted in Fig. 5.

To test whether intestinal sEH (encoded by *EPHX2*) is involved in the metabolism of 5(6)-EET, the stable *EPHX2* knockout (KO) LoVo cell line was constructed using CRISPR-Cas9 technology. *EPHX2* knockout plasmids (the knockout sequences are shown in the Table S5) and the vector plasmid (used as control) were purchased from Bio-rabbit (China, Shanghai). According to the manufacturer's instructions, lentivirus was packaged in HEK 293 T cells using the Lipofectamine 3000 Transfection Reagent (Invitrogen, #L3000008). Then, 48 h after the infection, the supernatant of HEK 293 T cells was collected to infect LoVo cells with polybrene to assist transfection. At last, the transfected cells were treated with puromycin to generate *EPHX2*-knockout stable cell lines. The knockout efficiency was evaluated by western blot (WB)

analysis (Fig. S2). The cells were then seeded in 24-well culture plates, and when the cell density was approximately 70% to 80%, 5(6)-EET or 14(15)-EET were added to NC cells, *EPHX2* KO cells, and cell-free medium, respectively, with the theoretical medium concentration of 2 μM. Then, 20 µl of the cell supernatant was collected at 0 h, 10 min, 30 min, 2.5 h, 5 h, and 10 h post-administration of EET, respectively. The collected sample was immediately transferred into a clean Eppendorf tube containing acetonile (70 μL) and internal standard (10 uL). After strong mixing for 2 min, the mixture was centrifuged at ×15000 g for 5 min under 4°C. The supernatant (50 μL) was transferred into a 150 μL-vial fixed in a 2 mL vial for analysis according to the previously reported method (4) with a modification of the LC running time to 12 min. Specifically, mobile phase A (A) comprised water with 0.1% acetic acid (v/v), and mobile phase B (B) comprised ACN/methanol/acetic acid (860/140/ 1, v/v/v). Gradient condition was: 0–0.25 min, 33% B; 0.25–0.5 min, 33%–45% B; 0.5–1.5 min, 45%–55% B; 1.5–4.5 min, 55%–60.8% B; 4.5–6.5 min, 60.8%–63% B; 6.5–7 min, 63%–73% B; 7–7.5 min, 73%–95% B; 7.5–8.5 min, 95% B; 8.5–9 min, 95%-33% B; 9–12 min, 33% B. A 10 µl aliquot of each sample was injected for analysis. The flow rate was 0.4 ml/min, and the column temperature was kept at 50◦C. Analytes were monitored by a SCIEX Triple Quad^{TM} 6500+ QTRAP (Framingham, MA) in a negative electrospray ion mode with a scheduled multiple reaction monitoring (MRM) mode. The parameters were the same as those in the reported method (4). Quality controls were performed with acceptable accuracy and precision.

Measurement of colon levels of lipid signaling molecules

The analysis of the lipid signaling molecules (SLMs) in colon tissue was conducted according to the previously reported method (4). The extraction procedure was modified. Specifically, the mouse colon tissue (~30 mg) was ground in a mortar with liquid nitrogen. The tissue slurry was

then completely transferred to a clean Eppendorf tube for solid phase extraction as described previously (4). The prepared samples were separated on a ZORBAX Eclipse Plus C_{18} column (2.1 × 150 mm, 1.8 μm) equipped with an Agilent 1290 II system (Santa Clara, CA). The eluting program was the same as that in the previous method (4). The separated SLMs were monitored by a SCIEX Triple Quad^{TM} 6500+ QTRAP (Framingham, MA) in a negative electrospray ion mode. The curtain gas, ion source gas 1 and 2, as well as the electrospray voltage and ion spray source temperature, were the same as the reported parameters in (4). The analytes were detected using scheduled multiple reaction monitoring (MRM) with the detailed parameters for each analyte was presented in (4). Quality controls were conducted with acceptable accuracy and precision.

qPCR analysis

The colon tissue and harvested cells were prepared for qPCR analysis following the manufacturer's instructions. The prepared samples were analyzed according to the methods reported previously (5). The primer sequences of target genes were presented in *SI Appendix* Table S1.

Western blot analysis

The colon tissue and harvested cells were prepared for immunoblot analysis of Claudin-1, Claudin-5, sEH, and β-Actin according to the manufacturer's instructions. Densitometric measurements of western blot results were conducted by using the software Image J (Image Processing and Analysis in Java). Optical density was normalized to β-Actin.

Statistical analysis

Data are presented as mean ± sd unless other noted. Statistical analyses were conducted by two-tailed *t*-test, or ANOVA followed by Tukey's (variance homogeneity) or Games-Howell's

(variance heterogeneity) post *ad hoc* comparison test using the software SPSS 22.0 (SPSS Inc., Chicago, IL) with $P < 0.05$ as the significant level. Orthogonal partial least squares discriminant analysis (OPLS-DA) and S-plot analysis were conducted by using SIMCA 14.1 (Umetrics, Sweden).

References

1. Hwang SH, Tsai HJ, Liu JY, Morisseau C, & Hammock BD (2007) Orally bioavailable potent soluble epoxide hydrolase inhibitors. *J Med Chem* 50(16):3825-3840.
2. Liu JY, Tsai HJ, Hwang SH, Jones PD, Morisseau C, & Hammock BD (2009) Pharmacokinetic optimization of four soluble epoxide hydrolase inhibitors for use in a murine model of inflammation. *Brit J Pharmacol* 156(2):284-296.
3. Luo Y, Wu MY, Deng BQ, Huang J, Hwang SH, Li MY*, et al.* (2019) Inhibition of soluble epoxide hydrolase attenuates a high-fat diet-mediated renal injury by activating PAX2 and AMPK. *P Natl Acad Sci USA* 116(11):5154-5159.
4. Fu X, Yin HH, Wu MJ, He X, Jiang Q, Zhang LT*, et al.* (2022) High Sensitivity and Wide Linearity LC-MS/MS Method for Oxylipin Quantification in Multiple Biological Samples. *J Lipid Res* 63(12):100302.
5. Luo Y, Wang L, Peng A, & Liu JY (2019) Metabolic profiling of human plasma reveals the activation of 5-lipoxygenase in the acute attack of gouty arthritis. *Rheumatology* 58(2):345-351.

Table S1 Primer sequences for real-time quantitative PCR

Gene	Species	Forward (5′ to 3′)	Reverse (5′ to 3′)
β-Actin	Mouse	TAGCCACCTTCCAGCAGATGT	AGCTCAGTAACAGTCCGCCTA
Claudin-1	Mouse	TCTACGAGGGACTGTGGATG	TCAGATTCAGCTAGGAGTCG
Claudin-5	Mouse	TTAAGGCACGGGTAGCACTC	GCACCGTCGGATCATAGAAC
Cyp2c29	Mouse	GCTCTCCTACTCCTGCTGAAGT	ATGTGGCTCCTGTCTTGCATGC
Cyp2c37	Mouse	AATGGAATGGGCCTTGCA	GCAACGTGCTTCTTCTTGAACG
Cyp2c38	Mouse	CACGGCCCATTGTTGTATTGC	TGAGTGTGAAACGTCTTGTCTCT
Cyp2c40	Mouse	GGCTCACAGCCTATTGTGGTA	TCAAAAACCGGAATCCTTCCTC
Cyp2c44	Mouse	GCTGCCCTATACAGATGCCG	GTGACGCTAAGAGTTGCCCA
Cyp2c50	Mouse	ACTGTGGTGTTGCATGGATATG	GAGAAGCGCCTTGTGTTTTTC
Cyp2c54	Mouse	AGACAGAGCTATGAAAGAGGGAA	GTGAGAAGTGCCTCGTGTTTT
Cyp2c55	Mouse	AATGATCTGGGGGTGATTTTCAG	GCGATCCTCGATGCTCCTC
Cyp2c65	Mouse	TCTGGGAAGCACTCCATCTCA	CCCTGGTGGGTAGTTTTTGG
Cyp2c68	Mouse	TTAGCCACGATCTGGGCAG	CTGGGGGATAGTTCTTGGGG
Cyp2c69	Mouse	ACAACCCAAAGACGGAGTTTAC	CTGTCCCAGCAGCAAACAGAT
Cyp2c70	Mouse	AGTATGGCCCTGTGTTTACTGT	GCCTTGGCTGGTTCTACTGAG
Cyp2j5	Mouse	TCTGGGAAGCACTCCATCTCA	CCCTGGTGGGTAGTTTTTGG
Cyp2j6	Mouse	TTAGCCACGATCTGGGCAG	CTGGGGGATAGTTCTTGGGG
Cyp2j8	Mouse	GCTACTATAGGTTGCCTGGTG	CCCAAATGCAGATGTGACAC
Cyp2j9	Mouse	TGGCTGATTTCCTCAAAAACCG	ACTGCTGAAGGGATAGGTGGG
Cyp2j11	Mouse	TTGACTGATCTGCATAGGGACC	CTTCAGGCTCGGCTTCTCATT
Cyp2j12	Mouse	AAGGAGGCTGACTGTCTTGTGG	GACTGTCCTCATACTCAAAGCGC
Cyp3a13	Mouse	CTGCCTTTCTTGGGGACGAT	CCGTCATACAACCCCCACAT
Ephx2	Mouse	GCGTTCGACCTTGACGGAG	TGTAGCTTTCATCCATGAGTGGT
Il-1β	Mouse	GCAACTGTTCCTGAACTCACT	ATCTTTTGGGGTCCGTCAACT
Mcp-1	Mouse	CTTCTGGGCCTGCTGTTCA	CCAGCCTACTCATTGGGATCA
Il-10	Mouse	GCTCTTACTGACTGGCATGAG	CGCAGCTCTAGGAGCATGTG
β-ACTIN	Human	CTCCATCCTGGCCTCGCTGT	GCTGTCACCTTCACCGTTCC
IL-1β	Human	AGCCATGGCAGAAGTACCTG	TGAAGCCCTTGCTGTAGTGG
IL-6	Human	CCACACAGACAGCCACTCAC	TGATTTTCACCAGGCAAGTCT
CLDN1	Human	CTGCCCCAGTGGAGGATTTA	CATGGCCTGGGCGGT
CLDN5	Human	CTCTGCTGGTTCGCCAACAT	CAGCTCGTACTTCTGCGACA

Table S2 Colorectum levels of oxylipins for the mice fed with an HSD and LSD （to be continued）

Oxylipins	LSD (nmol/kg, n = 8)		HSD (nmol/kg, n = 8)		*P* value*
	Mean	SD	Mean	SD	
13-HOTrE	158.0	103.2	89.4	30.0	0.1291
13-oxo-ODE	111.8	58.5	75.1	31.9	0.1729
9-oxo-ODE	101.6	44.3	74.8	53.6	0.3244
9(s)-HOTrE	23.6	10.7	13.7	4.0	0.0485
9(10)-EpOME	14.2	6.1	16.0	11.7	0.7285
13-HODE	2375.8	948.5	1378.7	591.1	0.0365
9-HODE	2067.4	744.2	1275.2	496.3	0.0369
12(13)-EpOME	14.3	6.3	16.4	12.7	0.7031
EKODE	39.2	15.7	84.9	86.5	0.2092
12,13-DiHOME	20.4	7.7	18.4	5.1	0.5740
9,10-DiHOME	19.7	6.1	19.4	4.5	0.9345
15-deoxy-$\Delta^{12,14}$-PGJ_2	0.2	0.2	0.3	0.1	0.3411
8-HEPE	1.2	0.6	0.7	0.3	0.0777
12-HEPE	29.0	16.2	16.9	5.7	0.0963
12-oxo-ETE	24.9	10.4	17.9	5.7	0.1445
15-oxo-ETE	26.4	4.4	22.7	3.2	0.0906
15-HEPE	27.0	8.3	18.4	8.1	0.0732
5-HEPE	1.7	0.4	1.2	0.6	0.1395
5-oxo-ETE	5.2	1.2	3.3	1.7	0.0256
9-HEPE	1.1	0.3	0.6	0.1	0.0020
11-HEPE	37.0	13.4	25.7	8.3	0.0813
20-HETE	1.8	1.3	1.6	1.1	0.7264
19(R)-HETE	1.1	0.3	0.9	0.6	0.5430
12-HETE	585.7	370.5	349.4	121.7	0.1453
5-HETE	35.0	7.0	31.3	12.6	0.5044
15-HETE	1200.4	324.6	1028.2	339.8	0.3487
11-HETE	630.4	247.9	510.3	168.0	0.3089
8-HETE	50.5	28.0	37.2	11.5	0.2737
9-HETE	20.5	5.1	18.3	5.6	0.4379
11(12)-EET	2.0	0.7	1.1	0.6	0.0164
14(15)-EET	3.6	0.9	2.6	1.6	0.1829
8(9)-EET	7.5	3.6	3.0	1.5	0.0125
5(6)-EET	102.1	57.3	31.4	17.1	0.0136

(Continued and to be continued)

Oxylipins	LSD (nmol/kg, n = 8)		HSD (nmol/kg, n = 8)		*P* value*
	Mean	SD	Mean	SD	
2,3-dinor-11β-$PGF_{2\alpha}$	2.5	1.2	3.3	1.5	0.2843
9,10,13-TriHOME	210.5	60.4	237.3	145.4	0.6627
9,12,13-TriHOME	248.3	83.2	263.0	155.4	0.8300
PGJ_2	25.8	14.9	22.3	6.2	0.5798
PGB_2	0.6	0.3	1.2	1.0	0.1461
5,15-DiHETE	5.1	2.0	4.5	2.6	0.6310
8,15-DiHETE	21.5	13.5	14.1	7.8	0.2353
17,18-DiHETE	2.6	0.8	1.7	0.6	0.0322
11,12-DiHETE	0.3	0.1	0.2	0.1	0.1249
6-trans-LTB_4	1.3	0.7	0.9	0.3	0.1427
11,12-DiHET	2.2	1.5	1.4	0.6	0.2433
14,15-DiHET	3.3	1.5	2.1	0.9	0.0988
8,9-DiHET	1.6	0.7	1.5	0.5	0.8204
5,6-DiHET	0.8	0.4	1.8	1.0	0.0564
19(20)-EpDPA	3.4	1.2	2.8	1.0	0.3246
13(14)-EpDPA	0.9	0.2	0.6	0.2	0.0719
7(8)-EpDPA	2.2	0.9	0.8	0.2	0.0040
22-HDHA	7.4	2.1	5.2	1.9	0.0568
4-HDHA	8.0	1.5	6.4	2.1	0.1234
8-HDHA	7.4	1.4	6.5	1.4	0.2885
14-HDHA	566.0	386.1	260.0	106.3	0.0777
10-HDHA	10.9	5.8	6.8	1.8	0.1101
16(17)-EpDPA	0.9	0.2	0.5	0.3	0.0085
10(11)-EpDPA	1.0	0.3	0.6	0.2	0.0042
7-HDHA	3.5	1.7	2.5	0.9	0.1763
13-HDHA	251.7	128.0	163.7	56.4	0.1281
16-HDHA	24.4	12.3	16.6	6.5	0.1643
20-HDHA	32.0	9.7	26.5	9.3	0.2929
17-HDHA	560.7	260.0	334.4	107.9	0.0612
11-HDHA	5.7	2.4	4.0	0.9	0.1190
PGD_3	12.1	7.6	10.5	5.9	0.6733
PGE_3	28.4	18.5	28.0	11.2	0.9627
13,14-didydro-15-keto PGE_2	39.3	18.1	53.6	35.3	0.3608

(Continued)

Oxylipins	LSD (nmol/kg, n = 8)		HSD (nmol/kg, n = 8)		*P* value*
	Mean	SD	Mean	SD	
13,14-dihydro-15-keto PGD_2	1.3	0.6	2.0	1.2	0.2068
PGE_2	780.1	357.2	889.3	420.8	0.6092
PGD_2	2327.1	1095.5	2240.4	1074.0	0.8833
LXA_4	2.0	0.7	1.6	0.5	0.2854
$PGF_{2\alpha}$	372.2	143.7	293.6	110.7	0.2718
PGD_1	487.6	283.7	332.0	122.7	0.2137
PGE_1	274.3	228.1	148.1	61.6	0.1952
Resolvin D_5	3.4	2.3	1.7	0.9	0.1075
16,17-DiHDPA	3.4	2.1	1.4	0.6	0.0462
13,14-DiHDPA	0.6	0.3	0.4	0.1	0.0908
19,20-DiHDPA	9.6	2.8	6.8	3.2	0.0991
10,11-DiHDPA	0.5	0.1	0.4	0.1	0.1944
Δ17-6-keto-$PGF_{1\alpha}$	27.3	13.4	29.3	14.7	0.7867
11-dehydro TXB_2	2.9	1.9	1.8	0.7	0.1914
6-keto-$PGF_{1\alpha}$	1834.0	725.9	2266.4	846.7	0.3228
TXB_2	239.6	92.8	319.1	154.3	0.2664
Resolvin D_1	0.5	0.1	0.4	0.2	0.5707

* statistical difference between the groups treated with LSD and HSD was determined by two-tailed *t*-student test.

DiHDPA: dihydroxydocosapentaenoic acid; DiHET: dihydroxyeicosatrienoic acid; DiHETE: dihydroxyeicosatetraenoic acid; DiHOME: dihydroxyoctadecenoic acid; EET: epoxyeicosatrienoic acid; EKODE: 12,13-epoxy-9-keto-10(trans)-octadecenoic acid; EpDPA: epoxydocosapentaenoic acid; EpOME: epoxyoctadecenoic acid; HDHA: hydroxydocosahexaenoic acid; HEPE: hydroxyeicosapentaenoic acid; HETE: hydroxyeicosatetraenoic acid; HOTrE: hydroxyoctadecatrienoic acid; LT: leukotriene; oxo-ETE: oxoeicosatetraenoic acid; oxo-ODE: oxooctadecadienoic acid; PG: prostaglandin; TriHOME: trihydroxyoctadecenoic acid; TX: thromboxane.

Table S3 Colorectum levels of oxylipins for the mice fed with an HSD, LSD, and LSD with TPPU （to be continued）

Oxylipins	LSD (nmol/kg, n=8)	HSD (nmol/kg, n=8)	HSD+TPPU (nmol/kg. n=8)	*P* values		
				LSD vs HSD	HSD vs HSD+TPPU	LSD vs HSD+TPPU
13-HOTrE	118.9 ± 77.3	65.4 ± 56.7	74.5 ± 36.8	0.0012	0.9498	0.0025
13-oxo-ODE	95.3 ± 59.0	52.9 ± 34.0	28.1 ± 13.7	0.2267	0.1899	0.0344
9-oxo-ODE	84.4 ± 64.5	42.2 ± 36.9	23.1 ± 9.9	0.2835	0.3814	0.0723
9(s)-HOTrE	20.4 ± 9.4	13.0 ± 21.7	6.6 ± 2.2	0.5372	0.6186	0.1311
9(10)-EpOME	34.8 ± 21.2	17.4 ± 8.5	21.0 ± 10.5	0.1319	0.7290	0.2719
13-HODE	2536 ± 1170	1165 ± 1526	785 ± 245	0.0579	0.7780	0.0135
9-HODE	1307 ± 565	1079 ± 1283	690 ± 232	0.8442	0.6173	0.3094
12(13)-EpOME	41.4 ± 21.0	19.8 ± 11.7	21.4 ± 11.3	0.0265	0.9742	0.0421
EKODE	42.1 ± 29.2	50.9 ± 36.8	19.7 ± 6.3	0.8588	0.1073	0.1480
12,13-DiHOME	17.6 ± 5.8	12.6 ± 17.9	8.0 ± 5.0	0.6545	0.6931	0.2251
9,10-DiHOME	17.4 ± 12.8	12.8 ± 15.2	9.0 ± 5.6	0.6188	0.7262	0.2239
15-deoxy-Δ12,14-PGJ2	0.45 ± 0.25	0.93 ± 0.95	1.02 ± 0.84	0.4037	0.9768	0.2227
8-HEPE	1.79 ± 0.74	0.76 ± 0.49	1.13 ± 0.60	0.0079	0.4540	0.1050
12-HEPE	35.0 ± 16.3	17.2 ± 8.6	32.3 ± 19.6	0.0474	0.1640	0.9505
12-oxo-ETE	34.2 ± 20.1	22.3 ± 18.6	26.9 ± 11.8	0.4336	0.8797	0.7225
15-oxo-ETE	19.5 ± 12.7	13.2 ± 10.6	12.1 ± 5.5	0.5398	0.9666	0.3300
15-HEPE	39.0 ± 21.7	17.5 ± 10.9	19.8 ±11.6	0.737	0.9189	0.1154
5-HEPE	3.10 ± 1.71	1.76 ± 0.82	2.25 ± 0.81	0.0864	0.6930	0.3476
5-oxo-ETE	5.77 ± 2.64	2.64 ± 1.35	5.74 ±4.05	0.1030	0.1061	0.9999
9-HEPE	2.78 ± 2.36	1.29 ± 2.25	0.70 ± 0.52	0.2853	0.8132	0.0992
11-HEPE	31.7 ± 16.3	25.4 ± 19.1	34.0 ± 14.6	0.7334	0.5657	0.9591
20-HETE	4.30 ± 2.20	3.97 ± 1.78	4.39 ± 2.75	0.9535	0.9281	0.9971

(Continued and to be continued)

Oxylipins	LSD (nmol/kg, n=8)	HSD (nmol/kg, n=8)	HSD+TPPU (nmol/kg. n=8)	*P* values		
				LSD vs HSD	HSD vs HSD+TPPU	LSD vs HSD+TPPU
19(R)-HETE	3.68 ± 2.59	2.77 ± 0.97	2.84 ± 1.03	0.6349	0.9867	0.6859
12-HETE	1725 ± 1175	875 ± 590	1270 ± 940	0.1703	0.7419	0.5040
5-HETE	76.8 ± 41.2	43.0 ± 24.8	59.0 ± 29.9	0.1210	0.5975	0.5314
15-HETE	1465 ± 660	1050 ± 990	1720 ± 560	0.5365	0.2117	0.7848
11-HETE	814 ± 373	564 ± 277	817 ± 326	0.3014	0.2915	0.9997
8-HETE	66.6 ± 23.1	47.7 ± 31.9	66.8 ± 35.8	0.4120	0.4454	0.9999
9-HETE	37.3 ± 22.0	24.5 ± 23.3	27.2 ± 15.0	0.4356	0.9609	0.5940
11(12)-EET	8.43 ± 5.69	3.09 ± 2.27	5.92 ± 1.44	0.0819	0.0284	0.4830
14(15)-EET	7.74 ± 2.47	4.61 ± 1.50	5.80 ± 2.79	0.0347	0.5724	0.2382
8(9)-EET	7.15 ± 3.72	4.30 ± 4.70	5.95 ± 8.79	0.6291	0.8541	0.9195
5(6)-EET	108.8 ± 17.1	46.9 ± 13.2	62.9 ± 23.1	<0.0001	0.2317	0.00016
2,3-dinor-11β-$PGF_{2\alpha}$	7.41 ± 4.52	8.26 ± 5.70	6.29 ± 2.47	0.9233	0.6548	0.8698
9,10,13-TriHOME	188.4 ± 90.9	151.0 ± 207.4	70.0 ± 28.8	0.8388	0.4497	0.1951
9,12,13-TriHOME	182.3 ± 53.8	151.0 ± 253.9	71.2 ± 34.3	0.9764	0.4312	0.3248
PGJ_2	37.8 ± 12.1	34.3 ± 31.0	50.9 ± 27.9	0.9571	0.3956	0.5569
PGB_2	1.28 ± 0.82	2.13 ± 2.60	1.11 ± 0.76	0.5661	0.4420	0.9756
5,15-DiHETE	7.62 ± 3.58	4.77 ± 3.51	4.75 ± 2.12	0.1875	0.9999	0.1840
8,15-DiHETE	31.1 ± 14.8	11.3 ± 7.0	17.4 ± 8.5	0.0167	0.3017	0.1022
17,18-DiHETE	4.16 ± 1.97	2.11 ± 0.78	1.81 ± 0.49	0.05293	0.6471	0.0276
11,12-DiHETE	0.66 ± 0.27	0.26 ± 0.26	0.23 ± 0.10	0.0052	0.9652	0.0028
6-trans-LTB_4	4.66 ± 3.88	2.79 ± 1.33	4.32 ± 2.11	0.3543	0.4945	0.9640
11,12-DiHET	3.28 ± 1.58	2.04 ± 0.97	1.86 ± 0.85	0.1108	0.9535	0.0627

(Continued and to be continued)

Oxylipins	LSD (nmol/kg, n=8)	HSD (nmol/kg, n=8)	HSD+TPPU (nmol/kg. n=8)	*P* values		
				LSD vs HSD	HSD vs HSD+TPPU	LSD vs HSD+TPPU
14,15-DiHET	5.23 ± 2.06	2.04 ± 0.97	1.86 ± 0.85	0.1112	0.9938	0.0909
8,9-DiHET	2.49 ± 1.16	1.54 ± 0.71	1.88 ± 0.84	0.1224	0.7439	0.3984
5,6-DiHET	0.96 ± 0.32	1.54 ± 0.68	0.94 ± 0.51	0.0914	0.0827	0.9985
19(20)-EpDPA	5.69 ± 2.25	3.02 ± 1.38	4.77 ± 2.18	0.0339	0.2020	0.6273
13(14)-EpDPA	1.28 ± 0.52	0.82 ± 0.29	1.17 ± 0.39	0.0810	0.2159	0.8503
7(8)-EpDPA	2.36 ± 0.77	0.83 ± 0.40	2.29 ± 0.91	0.0012	0.0052	0.9852
22-HDHA	7.61 ± 1.76	6.32 ± 3.08	7.05 ± 2.72	0.5879	0.8416	0.9021
4-HDHA	12.47 ± 5.73	8.79 ± 6.70	11.99 ± 6.57	0.4887	0.5790	0.9875
8-HDHA	13.37 ± 7.93	11.07 ± 8.50	11.73 ± 5.21	0.8080	0.9825	0.8965
14-HDHA	740 ± 280	500 ± 380	610 ± 340	0.3438	0.7930	0.7202
10-HDHA	16.67 ± 3.74	8.81 ± 4.50	16.21 ± 6.49	0.0139	0.0208	0.9817
16(17)-EpDPA	4.76 ± 2.54	2.48 ± 1.36	4.95 ± 1.67	0.0677	0.0457	0.9796
10(11)-EpDPA	3.06 ± 1.07	1.55 ± 0.81	3.30 ± 1.31	0.0288	0.0110	0.9005
7-HDHA	6.30 ± 2.36	3.32 ± 2.35	4.47 ± 2.27	0.0458	0.5888	0.2804
13-HDHA	121.6 ± 65.7	106.5 ± 64.4	131.3 ± 40.0	0.8616	0.6726	0.9401
16-HDHA	23.12 ± 14.71	12.99 ±8.55	20.90 ± 8.53	0.1804	0.3396	0.9145
20-HDHA	26.90 ± 8.84	20.47 ± 7.86	27.24 ± 12.85	0.4242	0.3888	0.9976
17-HDHA	574.8 ± 302.0	255.5 ± 189.1	258.0 ± 105.8	0.0189	0.9997	0.0199
11-HDHA	12.95 ± 5.86	7.36 ± 4.92	10.27 ± 5.04	0.1111	0.5255	0.5755
PGD_3	15.11 ± 6.38	12.60 ± 7.83	13.34 ± 7.15	0.7638	0.9763	0.8742
PGE_3	28.98 ± 19.53	27.81 ± 19.52	29.55 ± 11.31	0.9900	0.9779	0.9976
13,14-didydro-15-keto PGE_2	60.6 ±49.7	85.2 ± 43.0	71.8 ± 41.0	0.5227	0.8198	0.8722

(Continued)

Oxylipins	LSD (nmol/kg, n=8)	HSD (nmol/kg, n=8)	HSD+TPPU (nmol/kg. n=8)	*P* values		
				LSD vs HSD	HSD vs HSD+TPPU	LSD vs HSD+TPPU
13,14-dihydro-15-keto PGD_2	7.80 ± 3.06	6.50 ± 3.11	9.27 ± 5.72	0.6835	0.4758	0.8010
PGE_2	725 ± 360	910 ± 580	820 ± 255	0.7340	0.9169	0.8201
PGD_2	3035 ± 2195	3615 ± 2775	3015 ± 1130	0.8885	0.8410	0.9998
$PGF_{2\alpha}$	432.3 ± 124.9	310.9 ± 157.7	391.8 ± 106.7	0.1789	0.4491	0.8124
PGD_1	335.6 ± 137.4	347.9 ± 268.9	341.7 ± 111.3	0.9926	0.9980	0.9946
PGE_1	249.8 ± 114.5	182.6 ± 117.1	245.0 ± 106.8	0.4721	0.5217	0.9960
Resolvin D_5	4.37 ± 2.97	1.90 ± 1.36	2.70 ± 1.37	0.0615	0.7185	0.2525
16,17-DiHDPA	8.61 ± 3.10	2.26 ± 1.17	2.56 ± 1.30	< 0.0001	0.9526	<0.0001
13,14-DiHDPA	1.04 ± 0.44	0.63 ± 0.24	0.95 ± 0.77	0.2847	0.4441	0.9451
19,20-DiHDPA	14.68 ± 7.28	8.54 ± 2.75	9.05 ± 2.63	0.1188	0.9224	0.1557
10,11-DiHDPA	0.64 ± 0.36	0.36 ± 0.15	0.68 ± 0.58	0.3839	0.2819	0.9748
Δ17-6-keto-$PGF_{1\alpha}$	25.08 ± 16.73	26.71 ±19.32	24.59 ± 9.68	0.9766	0.9612	0.9979
11-dehydro TXB_2	4.70 ± 3.38	3.00 ± 2.98	4.79 ± 3.07	0.5339	0.5054	0.9987
6-keto-$PGF_{1\alpha}$	1940 ± 785	2370 ± 1550	2155 ±700	0.7079	0.9151	0.9172
TXB_2	216.4 ± 81.8	277.5 ± 213.8	236.7 ± 108.3	0.7378	0.8812	0.9066
Resolvin D_1	0.26 ± 018	0.18 ± 0.08	0.16 ± 0.06	0.5313	0.8524	0.3716

The statistical difference was determined by ANOVA followed by a Tukey's (variance homogeneity) or Dunnett's (variance heterogeneity) post hoc comparison test. The abbreviations were the same as those in Table S2.

Table S4 Colorectum levels of oxylipins for the icKO mice and WT littermates fed with an LSD or HSD （to be continued）

Oxylipins	WT (nmol/kg, n = 5 each)			icKO sEH (nmol/kg, n = 5)			*P* value	
	LSD (1)	HSD (2)	*P* value	LSD (3)	HSD (4)	*P* value	LSD (1 vs 3)	HSD (2 vs 4)
13-HOTrE	155.6 ± 65.2	44.5 ± 20.3	0.0559	338.1 ± 151.2	56.2 ± 28.4	0.0439	0.1704	0.8723
13-oxo-ODE	57.3 ±16.7	37.1 ± 7.5	0.1719	80.8 ± 32.5	28.2 ± 6.4	0.0700	0.5238	0.2650
9-oxo-ODE	49.9 ± 8.3	33.7 ± 7.9	0.0543	66.5 ± 39.3	25.1 ± 7.1	0.2295	0.7951	0.3340
9(s)-HOTrE	18.7 ± 4.9	5.9 ± 1.4	0.0009	23.8 ± 6.0	7.7 ± 2.6	0.0001	0.2486	0.9061
9(10)-EpOME	39.6 ± 12.4	37.6 ± 6.3	0.9932	45.4 ± 19.8	17.1 ± 8.5	0.0140	0.8886	0.0921
13-HODE	1955 ± 595	695 ± 210	0.0238	2940 ± 210	930 ± 275	< 0.0001	0.0618	0.4709
9-HODE	1970 ± 670	605 ± 155	0.0316	2415 ± 240	850 ± 165	< 0.0001	0.5453	0.1563
12(13)-EpOME	36.0 ± 8.4	37.0 ± 9.2	0.9657	51.0 ± 21.9	19.6 ± 9.4	0.0095	0.3245	0.1181
EKODE	33.6 ± 14.0	36.3 ± 9.4	0.9826	40.8 ± 24.1	23.9 ± 7.4	0.5020	0.9348	0.1768
12,13-DiHOME	23.6 ± 5.1	7.2 ± 0.8	< 0.0001	21.1 ± 2.2	7.74 ± 3.6	< 0.0001	0.6703	0.9955
9,10-DiHOME	20.6 ± 6.4	8.4 ± 0.9	0.0422	19.9 ± 3.2	8.2 ± 3.8	0.0036	0.9968	0.9983
15-deoxy-Δ12,14-PGJ2	2.67 ± 1.42	1.27 ±0.75	0.3059	3.49 ± 2.74	2.67 ± 1.78	0.9265	0.9301	0.2246
8-HEPE	0.94 ± 0.29	1.04 ± 0.66	0.9961	1.46 ± 0.64	1.31 ± 1.00	0.9870	0.6475	0.9210
12-HEPE	32.0 ± 5.9	21.8 ± 13.8	0.6867	41.8 ± 11.5	27.8 ± 21.8	0.4451	0.7116	0.9116
12-oxo-ETE	24.9 ± 7.7	25.0 ± 12.7	1.0000	27.8 ± 11.0	25.6 ± 14.4	0.9903	0.9793	0.9998
15-oxo-ETE	17.0 ± 3.9	13.6 ± 3.8	0.5561	18.7 ± 9.0	12.7 ± 3.7	0.5611	0.9785	0.9760
15-HEPE	41.1 ± 11.0	20.6 ± 12.5	0.1344	43.2 ± 10.8	23.8 ± 19.6	0.1666	0.9949	0.9826
5-HEPE	2.89 ± 0.64	3.17 ± 1.36	0.9856	2.87 ± 1.25	2.62 ± 1.77	0.9902	1.0000	0.9078
5-oxo-ETE	2.70 ± 0.43	3.64 ± 1.39	0.9315	4.09 ± 4.30	4.25 ± 2.06	0.9996	0.8155	0.9796
9-HEPE	0.51 ± 0.22	0.61 ± 0.43	0.9698	1.10 ± 0.24	0.79 ± 0.46	0.5398	0.0789	0.8474
11-HEPE	52.7 ± 14.9	26.6 ± 7.7	0.0140	57.6 ± 10.3	39.9 ± 12.8	0.1208	0.9088	0.3148
20-HETE	5.20 ± 3.32	4.06 ± 3.21	0.9428	4.06 ± 1.90	3.42 ± 0.59	0.8839	0.9060	0.9685

(Continued and to be continued)

Oxylipins	WT (nmol/kg, n = 5 each)			icKO sEH (nmol/kg, n = 5)			*P* value	
	LSD (1)	HSD (2)	*P* value	LSD (3)	HSD (4)	*P* value	LSD (1 vs 3)	HSD (2 vs 4)
19(R)-HETE	2.06 ± 0.82	2.76 ± 0.72	0.4367	2.25 ± 0.37	2.56 ± 0.86	0.9025	0.9734	0.9713
12-HETE	960 ± 150	480 ± 165	0.4705	1405 ± 495	1150 ± 875	0.8620	0.5420	0.2086
5-HETE	50.4 ± 10.1	64.0 ± 37.7	0.9068	68.6 ± 37.9	75.4 ± 34.7	0.9868	0.8070	0.9427
15-HETE	2160 ± 340	1370 ± 500	0.1382	2785 ± 650	2160 ± 625	0.2968	0.3005	0.1403
11-HETE	1245 ± 225	770 ± 280	0.0825	1625 ± 375	1250 ± 265	0.2201	0.2114	0.0787
8-HETE	44.9 ± 22.2	34.4 ± 28.0	0.9725	76.4 ± 33.0	69.5 ± 59.8	0.9918	0.5800	0.4920
9-HETE	15.2 ± 3.0	20.9 ± 8.6	0.7680	30.1 ± 11.6	26.1 ± 10.9	0.9009	0.0886	0.8051
11(12)-EET	10.4 ± 5.1	6.57 ± 3.39	0.5699	8.87 ± 3.94	8.75 ± 5.75	1.0000	0.9511	0.8782
14(15)-EET	36.3 ± 16.9	26.8 ± 20.0	0.7696	22.4 ± 7.7	22.5 ± 15.2	1.0000	0.5125	0.9721
8(9)-EET	9.40 ± 2.41	6.03 ± 2.26	0.3488	9.45 ± 4.13	9.16 ± 3.28	0.9988	1.0000	0.4115
5(6)-EET	153.4 ± 29.4	41.8 ± 16.6	< 0.0001	159.2 ± 35.8	83.9 ± 21.5	0.0021	0.9856	0.1031
2,3-dinor-11β-$PGF_{2\alpha}$	6.83 ± 3.38	6.98 ± 1.70	0.9998	10.91 ± 1.51	9.78 ± 1.54	0.6582	0.1717	0.0980
9,10,13-TriHOME	155.7 ± 80.7	67.3 ± 4.3	0.2087	149.4 ± 83.6	69.9 ± 16.2	0.2882	0.9993	0.9832
9,12,13-TriHOME	153.0 ± 74.9	55.4 ± 30.2	0.1004	169.7 ± 93.4	66.0 ± 12.6	0.0755	0.9734	0.9929
PGJ_2	64.0 ± 27.0	34.0 ± 14.6	0.3526	79.6 ± 25.6	72.4 ± 38.4	0.9760	0.8077	0.1679
PGB_2	1.43 ± 0.44	0.74 ± 0.48	0.1616	2.59 ± 1.14	2.03 ± 0.67	0.7820	0.2581	0.0387
5,15-DiHETE	4.03 ± 1.07	5.63 ± 2.89	0.7410	6.06 ± 2.35	5.83 ± 3.11	0.9989	0.5787	0.9991
8,15-DiHETE	12.3 ± 7.4	10.9 ± 5.9	0.9802	15.7 ± 6.0	11.8 ± 3.6	0.7364	0.8033	0.9936
17,18-DiHETE	1.83 ± 0.41	1.34 ± 0.14	0.2279	1.86 ± 0.41	1.52 ± 0.57	0.5859	0.9995	0.8951
11,12-DiHETE	0.16 ± 0.07	0.22 ± 0.12	0.8613	0.27 ± 0.10	0.27 ± 0.12	1.0000	0.4271	0.8753
6-trans-LTB_4	2.08 ± 0.77	1.90 ± 1.22	0.9962	3.92 ± 2.06	2.76 ± 0.82	0.5210	0.1636	0.7337
11,12-DiHET	1.23 ± 0.45	1.23 ± 0.28	1.0000	1.86 ± 0.49	1.36 ± 0.29	0.2194	0.0916	0.9571

(Continued and to be continued)

Oxylipins	WT (nmol/kg, n = 5 each)			icKO sEH (nmol/kg, n = 5)			*P* value	
	LSD (1)	HSD (2)	*P* value	LSD (3)	HSD (4)	*P* value	LSD (1 vs 3)	HSD (2 vs 4)
14,15-DiHET	2.57 ± 0.16	2.09 ± 0.45	1.000	2.37 ± 0.56	1.96 ± 0.42	0.2194	0.0916	0.9571
8,9-DiHET	0.93 ± 0.19	1.14 ± 0.47	0.6408	1.16 ± 0.16	0.82 ± 0.14	0.2310	0.5547	0.2878
5,6-DiHET	0.62 ± 0.17	0.68 ± 0.13	0.9658	1.08 ± 0.31	0.73 ± 0.29	0.1375	0.0319	0.9882
19(20)-EpDPA	12.4 ± 2.55	11.67 ± 3.45	0.9822	8.74 ± 2.76	9.40 ± 4.08	0.9883	0.3177	0.6991
13(14)-EpDPA	5.29 ± 1.48	5.89 ± 2.88	0.9746	4.08 ± 1.65	4.71 ± 2.66	0.9702	0.8295	0.8409
7(8)-EpDPA	4.30 ± 1.22	2.76 ± 0.95	0.5585	4.57 ± 1.93	6.55 ± 2.70	0.3517	0.9954	0.0224
22-HDHA	11.2 ± 4.1	10.9 ± 5.4	0.9992	11.1 ± 1.4	10.4 ± 2.2	0.9888	1.0000	0.9968
4-HDHA	7.28 ± 0.88	6.23 ± 1.27	0.4785	14.5 ± 11.0	12.8 ± 4.1	0.9867	0.5228	0.0670
8-HDHA	6.77 ± 0.93	6.06 ± 1.78	0.8540	13.2 ± 10.2	10.7 ± 3.6	0.9536	0.5592	0.1362
14-HDHA	495 ± 140	265 ± 120	0.0916	605 ± 180	445 ± 130	0.3368	0.6444	0.2350
10-HDHA	10.1 ± 1.9	6.20 ± 2.82	0.5025	16.8 ± 6.90	14.7 ± 4.0	0.8766	0.1095	0.0304
16(17)-EpDPA	6.63 ± 1.99	3.92 ± 1.80	0.2357	5.73 ± 2.47	5.97 ± 2.34	0.9981	0.9111	0.4634
10(11)-EpDPA	4.71 ± 1.38	2.41 ± 0.40	0.0617	5.04 ± 2.13	3.07 ± 1.31	0.3673	0.9907	0.7127
7-HDHA	1.99 ± 0.53	1.66 ± 0.50	0.7508	4.32 ± 3.06	3.93 ± 1.52	0.9934	0.4336	0.0889
13-HDHA	210.9 ± 50.6	132.2 ± 58.0	0.0949	255.6 ± 46.3	207.3 ± 40.7	0.4335	0.4979	0.1166
16-HDHA	27.1 ± 6.2	20.2 ± 9.7	0.6016	42.9 ± 10.8	33.1 ± 7.6	0.3219	0.0511	0.1315
20-HDHA	50.0 ± 7.2	43.6 ± 19.7	0.9368	81.6 ± 24.4	64.3 ± 13.2	0.4209	0.0495	0.2776
17-HDHA	388.7 ± 139.1	166.1 ± 49.2	0.0690	375.2 ± 87.1	280.1 ± 189.4	0.7456	0.9975	0.6625
11-HDHA	3.86 ± 0.42	2.90 ± 1.53	0.5808	6.50 ± 1.38	6.48 ± 2.42	1.0000	0.0372	0.1019
PGD_3	58.2 ± 20.3	45.9 ± 13.8	0.5256	53.2 ± 9.32	49.9 ± 9.8	0.9815	0.9430	0.9680
PGE_3	31.1 ± 9.6	29.2 ± 8.0	0.9864	37.3 ± 6.3	33.5 ± 11.8	0.9123	0.7132	0.8757
13,14-didydro-15-keto PGE_2	43.9 ± 22.2	67.1 ± 18.8	0.3650	44.7 ± 14.4	59.8 ± 29.0	0.6953	0.9999	0.9518

(Continued)

Oxylipins	WT (nmol/kg, n=5 each)			icKO sEH (nmol/kg, n=5)			*P* value	
	LSD (1)	HSD (2)	*P* value	LSD (3)	HSD (4)	*P* value	LSD (1 vs 3)	HSD (2 vs 4)
13,14-dihydro-15-keto PGD_2	4.69 ± 2.11	7.79 ± 2.46	0.1608	6.24 ± 2.66	8.94 ± 1.36	0.2518	0.6891	0.8390
PGE_2	975 ± 320	1225 ± 165	0.4181	945 ± 255	765 ± 230	0.6711	0.9973	0.0453
PGD_2	3165 ± 845	3255 ± 850	0.9961	4355 ± 240	3905 ± 550	0.7206	0.0556	0.4428
$PGF_{2\alpha}$	565 ± 180	425 ± 245	0.7732	565 ± 240	510 ± 240	0.9759	1.0000	0.9418
PGD_1	400 ± 140	210 ± 90	0.0595	360 ± 125	275 ± 50	0.5952	0.9504	0.7671
PGE_1	330 ± 110	205 ± 100	0.2278	380 ± 110	275 ± 80	0.3771	0.8633	0.6805
Resolvin D_5	1.47 ± 0.78	1.21 ± 0.93	0.9650	2.77 ± 1.11	1.50 ± 0.43	0.1254	0.1131	0.9514
16,17-DiHDPA	3.83 ± 1.69	2.55 ± 0.82	0.3749	4.80 ± 1.27	2.64 ± 0.90	0.0568	0.6032	0.9993
13,14-DiHDPA	0.51 ± 0.15	0.48 ± 0.20	0.9971	0.76 ± 0.27	0.62 ± 0.26	0.7554	0.3420	0.7870
19,20-DiHDPA	10.23 ± 2.33	6.98 ± 2.30	0.1073	12.10 ± 2.14	6.95 ± 1.56	0.0067	0.5158	1.0000
10,11-DiHDPA	0.38 ± 0.18	0.31 ± 0.11	0.8828	0.54 ± 0.16	0.40 ± 0.17	0.5434	0.3916	0.7487
Δ17-6-keto-$PGF_{1\alpha}$	29.0 ± 12.7	19.6 ± 5.4	0.4662	30.7 ± 12.7	23.0 ± 6.8	0.6197	0.9926	0.9491
11-dehydro TXB_2	9.98 ± 2.91	8.12 ± 6.42	0.8932	9.31 ± 1.71	8.90 ± 4.05	0.9985	0.9940	0.9909
6-keto-$PGF_{1\alpha}$	2205 ± 650	2825 ± 860	0.5791	3055 ± 1400	3890 ± 1040	0.6149	0.6328	0.1650
TXB_2	255.9 ± 90.8	220.2 ± 52.8	0.8698	377.9 ± 118.4	236.9 ± 34.9	0.1707	0.3318	0.9323
Resolvin D_1	0.46 ± 0.47	0.18 ± 0.05	0.5872	0.30 ± 0.08	0.24 ± 0.12	0.8166	0.8686	0.7005

The statistical difference was determined by ANOVA followed by a Tukey's (variance homogeneity) or Dunnett's (variance heterogeneity) post hoc comparison test. The abbreviations were the same as those in Table S2.

Table S5 The sequences of EPHX2 knockout plasmids used in this project

	Forward (5′ to 3′)	Reverse (5′ to 3′)
sg*EPHX2*-1	CACCGCCTGATAGAGTCGTGTCAGG	AAACCCTGACACGACTCTATCAGGC
sg*EPHX2*-2	CACCGGAGGCAGACTTTAGCGGTCT	AAACAGACCGCTAAAGTCTGCCTCC
sg*EPHX2*-3	CACCGTACATCTTTGAGACCACCGG	AAACCCGGTGGTCTCAAAGATGTAC

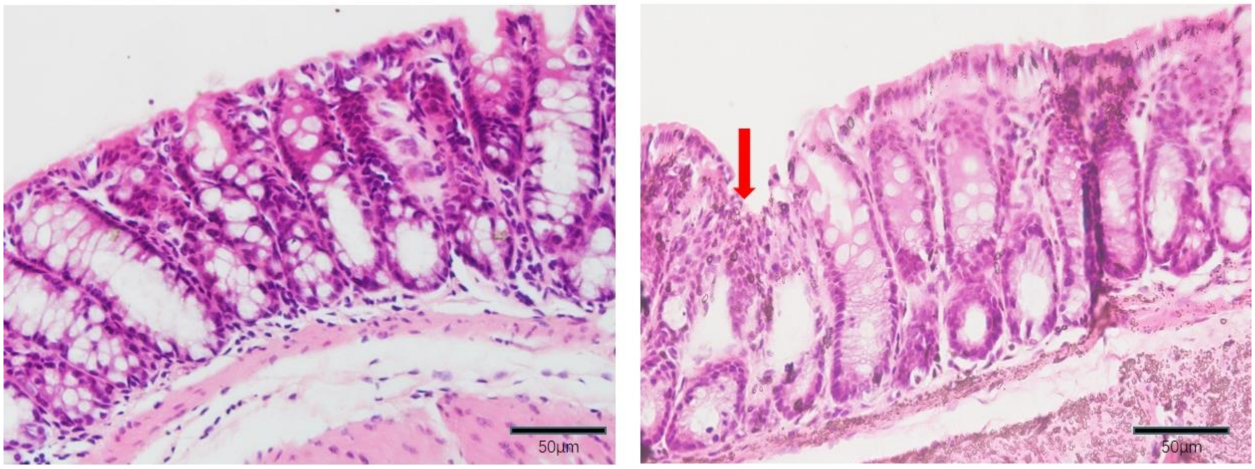

Fig. S1 Sixteen weeks of HSD feeding resulted in inflammatory cell infiltration in murine colon tissue. The representative photomicrographs of colon tissue from the mice fed with an LSD (left) and an HSD (right) for 16 weeks. Tissue slices were stained with hematoxylin and eosin (H.&E.). Photomicrographs are shown at 400× magnification. The red arrow indicates the inflammatory cell infiltration. The scale marked in the right bottom corner represents 50 µm.

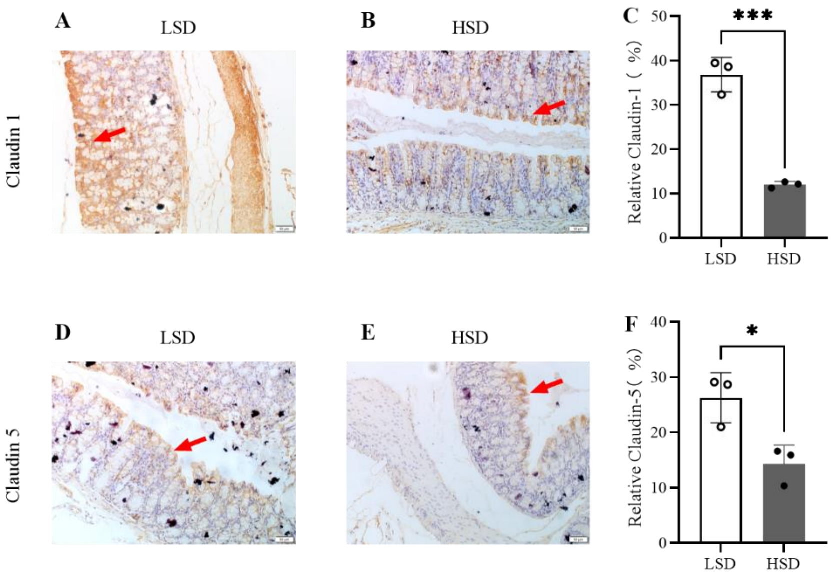

Fig. S2 Sixteen weeks of HSD feeding caused a decrease in intestinal Claudin 1 and Caudin-5. Representative IHC images of Claudin 1 (**A** and **B**) and Claudin 5 (**D** and **E**) expression in of mouse colon sections (stained in brown as pointed by red arrow indicate Claudin 1 or 5, scale bar = 50 μm); Statistical analyses of protein expression of Claudin 1 (**C**) and Claudin 5 (**F**). Data represent mean ± SD (n = 3). * Statistical difference (0.01< p ≤ 0.05), and *** statistical difference (0.0001< p ≤ 0.001) between LSD and HSD groups was determined by a two-tailed *t*-student test.

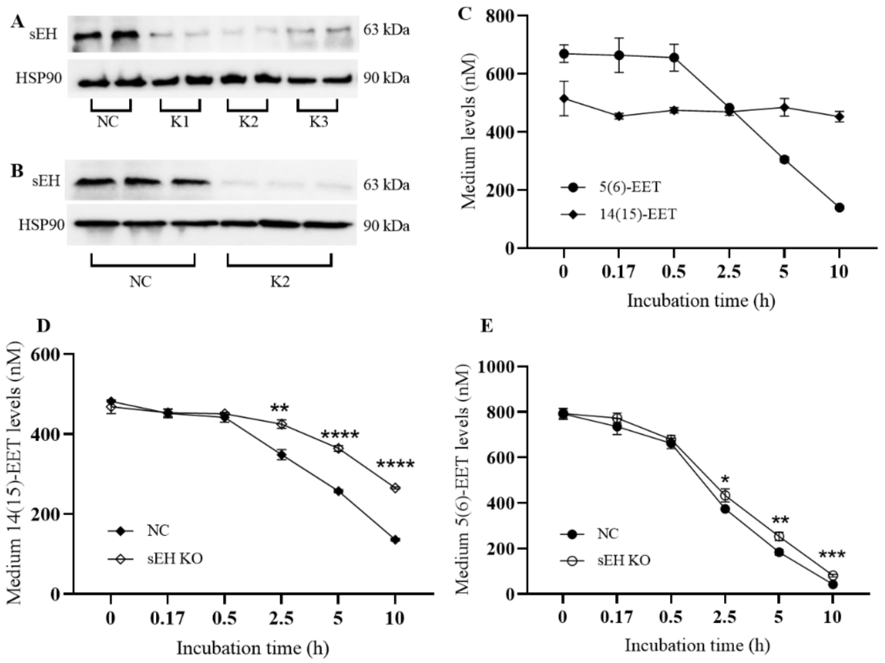

Fig.S3 Soluble epoxide hydrolase is involved in the metabolism of 5(6)-EET. (**A**) The plasmid K2 showed the best efficiency in knockout of sEH; (**B**) LoVo cells with knockout of sEH by the plasmid K2 was successfully established; (**C**) 5(6)-EET was degraded in cell media along the time (black dots and line) while 14(15)-EET kept stable during the tested time (black diamonds and line); (**D**) When within the cellular system, 14(15)-EET was metabolized by enzymes (filled diamonds and line) but the metabolism was slowed down by knockout of sEH (unfilled diamonds and line), indicating 14(15) was metabolized partially by sEH; (**E**) Within a cellular system, the metabolism of 5(6)-EET was also slowed down by knockout of sEH, indicating the metabolism of 5(6)-EET is mediated partially by sEH. Data represent mean ± SD (n = 3). * Statistical difference ($0.01 < p \leqslant 0.05$), ** statistical difference ($0.001 < p \leq 0.01$), and *** statistical difference ($0.0001 < p \leq 0.001$) between NC cells and sEH knockout cells at the same time point determined by two-tailed *t*-student test.

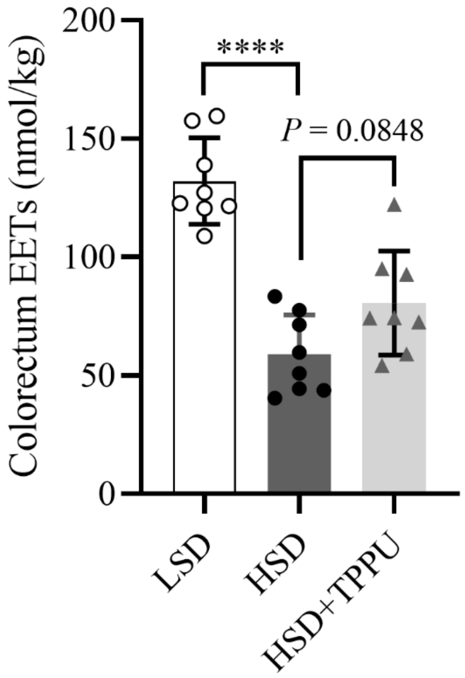

Fig. S4 TPPU treatment attenuated the HSD-mediated decrease in colorectum EETs. Here the EETs are the sum of 14(15)-, 11(12)-, 8(9)- and 5(6)-EET. Statistical difference was determined by ANOVO followed by Tukey's comparison (****, $p < 0.0001$). The colorectum concentration of specific EET was presented in Table S3.

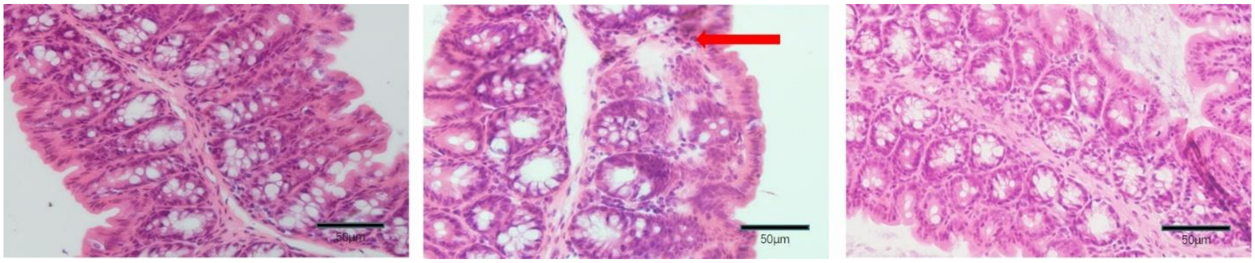

Fig. S5 The treatment of *t*-TUCB attenuated HSD-mediated inflammatory cell infiltration in murine colon tissue. The representative photomicrographs of colon tissue from the mice fed with an LSD (left), HSD (middle), and HSD with *t*-TUCB (right) for 16 weeks. Tissue slices were stained with hematoxylin and eosin (H.&E.). Photomicrographs are shown at 400× magnification. The red arrow indicates the inflammatory cell infiltration. The scale marked in the right bottom corner represents 50 μm.

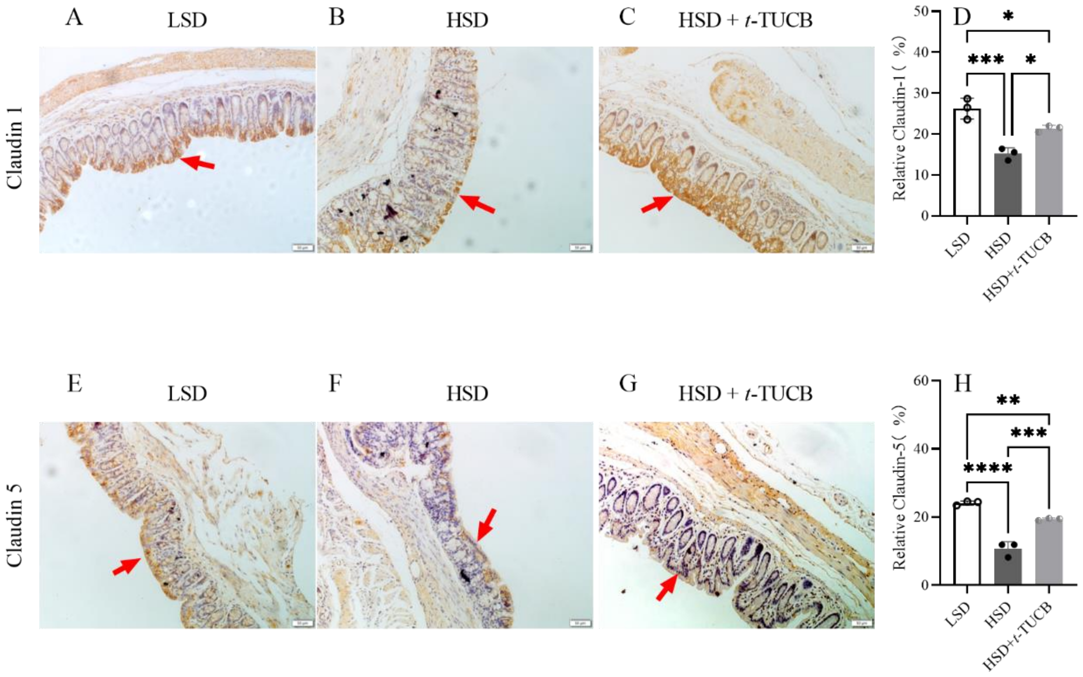

Fig. S6 The treatment of *t*-TUCB restored HSD-caused decrease in colon Claudin 1 and Claudin 5. Representative IHC images of Claudin 1 (**A**, **B**, and **C**) and Claudin 5 (**E**, **F**, and **G**) expression in of mouse colon sections (stained in brown as pointed by red arrow indicate Claudin 1 or Claudin 5, scale bar = 50 μm); Statistical analyses of protein expression of Claudin 1 (**D**) and Claudin 5 (**H**) . Data represent mean ± SD (n = 3). * Statistical difference ($0.01< p \leqslant 0.05$), ** statistical difference ($0.001< p \leqslant 0.01$), *** statistical difference ($0.0001< p \leqslant 0.001$), and **** statistical difference ($p \leqslant 0.0001$) between indicated groups was determined by ANOVA followed by Tukey's comparison.

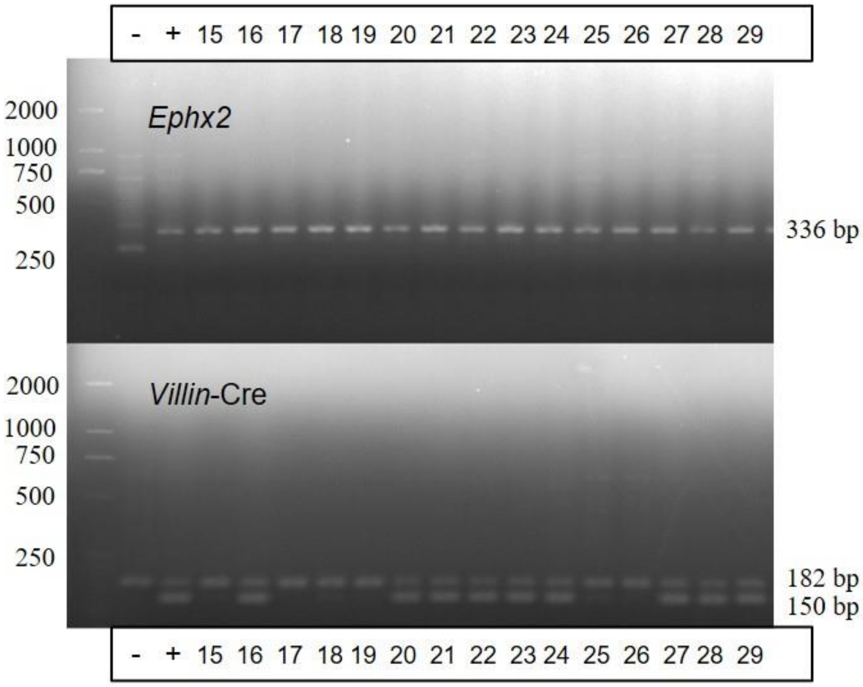

Fig. S7 The representative PCR identification of *Ephx2*$^{flox/flox}$; Villin-Cre mouse (cKO) and its wildtype (WT). Here the mice of 16, 20, 21, 22, 23,24, 27, 28, and 29 were cKO mice and 15, 17, 18, 19, 25,and 26 were WT mice.

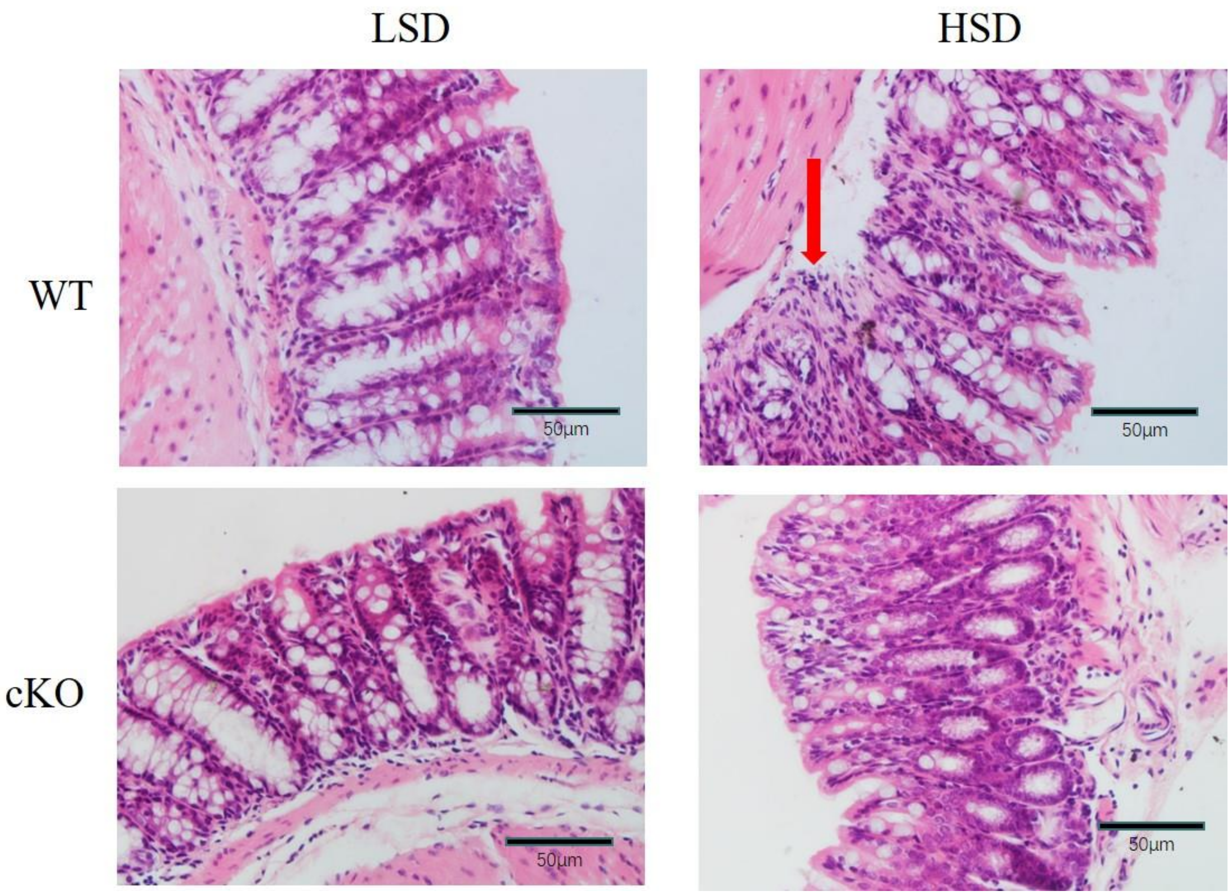

Fig. S8 Intestinal epithelial-specific knockout (cKO) of soluble epoxide hydrolase attenuates HSD-mediated injury to colon tissue. The representative photomicrographs of colon tissue from the WT mice fed with an LSD (top left) and HSD (top right), and the cKO mice fed with an LSD (bottom left) and HSD (bottom right) for 16 weeks. Tissue slices were stained with hematoxylin and eosin (H.&E.). Photomicrographs are shown at 400× magnification. The red arrow indicates the inflammatory cell infiltration. The scale marked in the right bottom corner represents 50 μm.

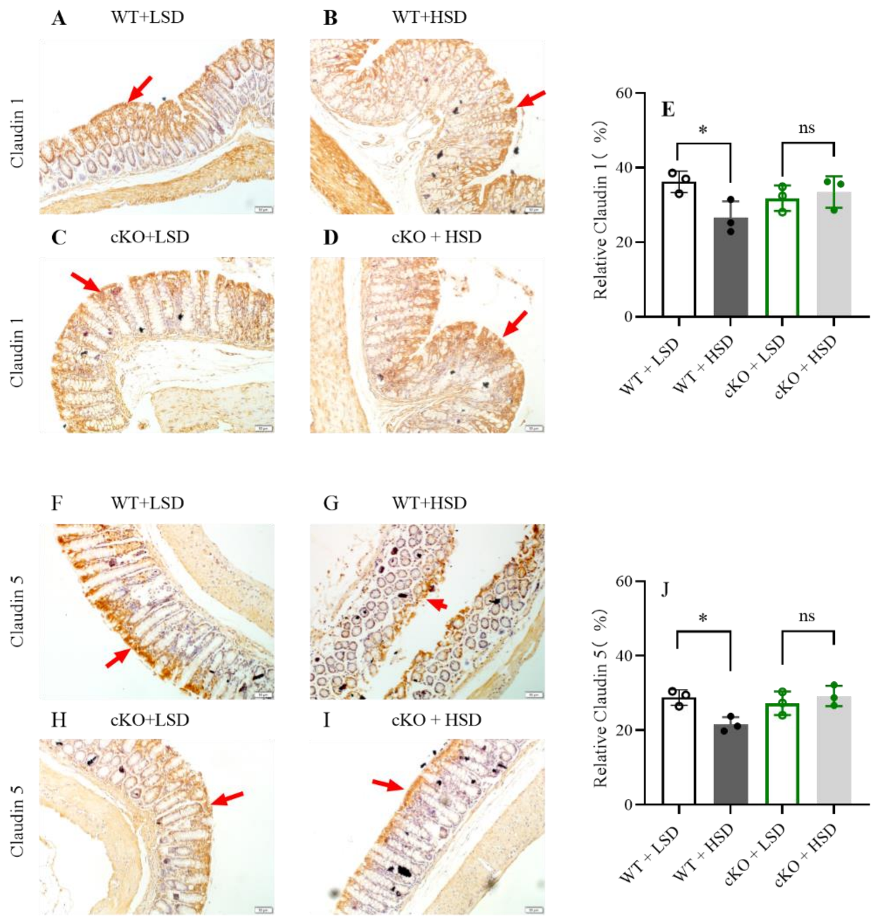

Fig. S9 HSD feeding caused a decrease in intestinal Claudin 1 and Caudin-5 for wildtype mice (WT) while didn't affect the mice with intestinal epithelial conditional knockout of sEH (cKO). Representative IHC images of Claudin 1 (**A** - **D**) and Claudin 5 (**F** and *I*) expression in of mouse colon sections (stained in brown as pointed by red arrow indicate Claudin 1 or 5, scale bar = 50 μm); Statistical analyses of protein expression of Claudin 1 (**E**) and Claudin 5 (**J**). Data represent mean ± SD (n = 3). * Statistical difference ($0.01 < p \leqslant 0.05$) between LSD and HSD groups was determined by a two-tailed *t*-student test.

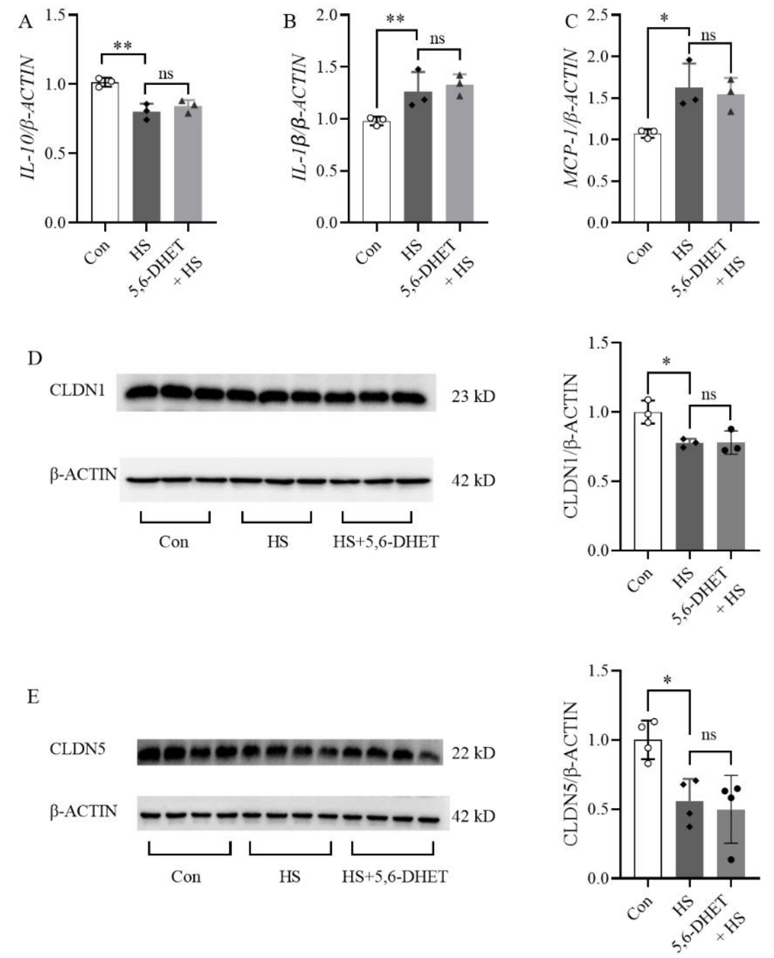

Fig. S10 5,6-DiHET failed in reversing high sucrose (HS)-induced damages to intestinal epithelial cells. 5,6-DiHET (100 nM) failed in reversing HS-induced decrease in *IL-10* (**A**), Claudin 1 (**D**), and Claudin 5 (**E**), and HS-induced increase in *IL-1β* (**B**) and *Mcp-1* (**C**). Data represent mean ± SD (N = 3-4). Statistical difference was determined by ANOVA followed by a Bonferroni's (variance homogeneity) or Dunnett's (variance heterogeneity) post hoc comparison test (ns, nonsignificant, * $0.01 < P \leqslant 0.05$, **$0.001 < P \leqslant 0.01$).

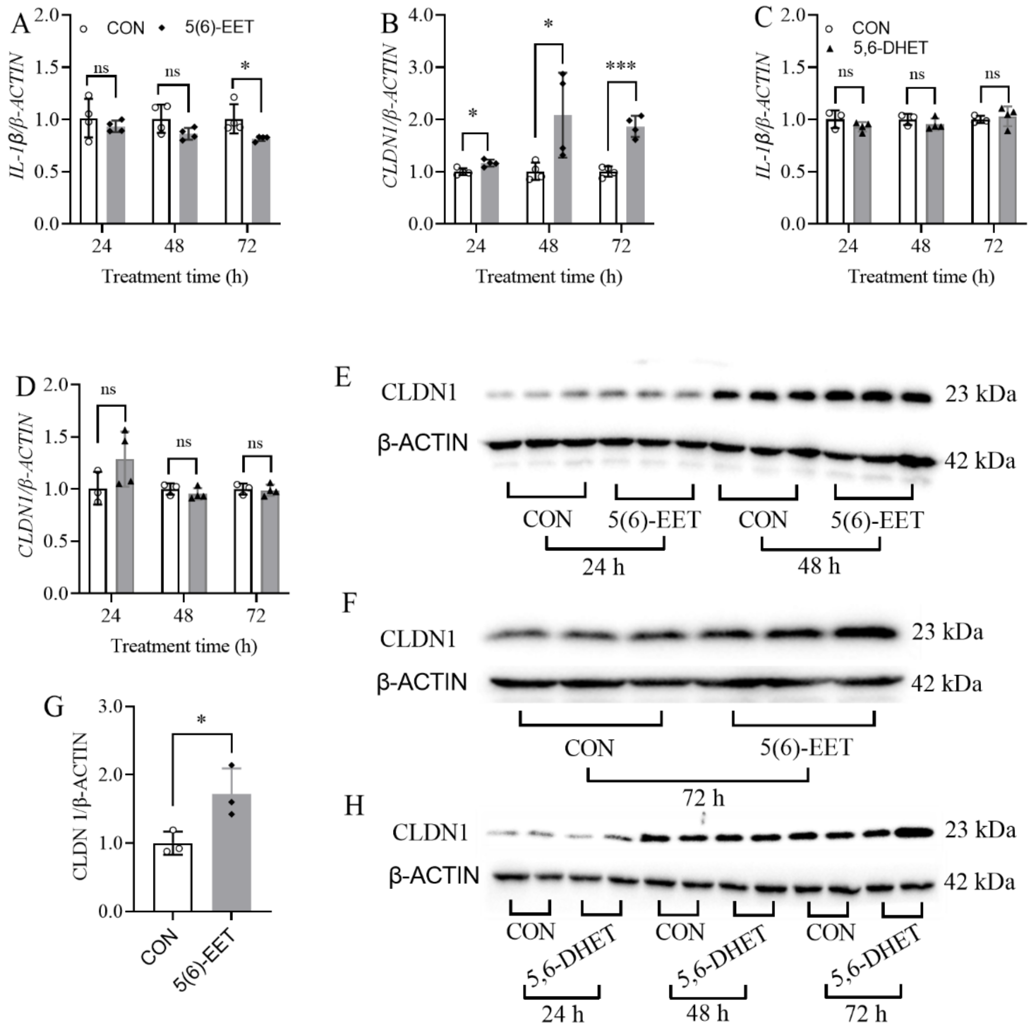

Fig.S11 5(6)-EET but not 5,6-DiHET is anti-inflammatory and increases tight junction intestinal epithelia in a time-relevant manner. Treatment of 5(6)-EET time-dependently reduced cellular *IL-1β* (**A**) and increased CLAUDIN 1 at mRNA (**B**) and protein (**E**, **F**, and **G**) levels. Treatment of 5,6-DiHET slightly impacts cellular *IL-1β* (**C**) and CLAUDIN 1 at mRNA (**D**) and protein (**H**) levels. Data represent mean ± SD (N = 3-4). Statistical difference between the two groups was determined by a two-tailed noncompartmental test with the Mann-Whitney test (* $0.01 < P \leqslant 0.05$, ***$0.0001 < P \leqslant 0.001$).

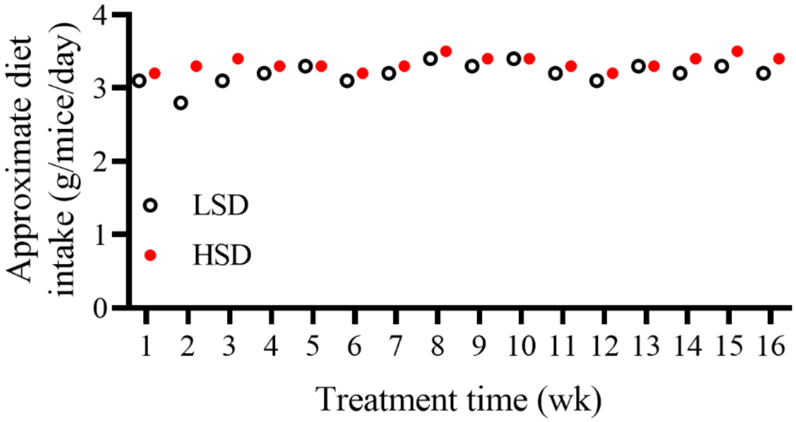

Fig. S12 Approximate average diet intake calculated by the diet consumption per cage every week. Data represent the mean of the data calculated from two cages with no obviously difference.
